# Distinct spatial distribution and roles of Kupffer cells and monocyte-derived macrophages in mouse acute liver injury

**DOI:** 10.3389/fimmu.2022.994480

**Published:** 2022-09-30

**Authors:** Manuel Flores Molina, Mohamed N. Abdelnabi, Sabrina Mazouz, Deborah Villafranca-Baughman, Vincent Quoc-Huy Trinh, Shafi Muhammad, Nathalie Bédard, David Osorio Laverde, Ghada S. Hassan, Adriana Di Polo, Naglaa H. Shoukry

**Affiliations:** ^1^ Centre de Recherche du Centre hospitalier de l’Université de Montréal (CRCHUM), Montréal, QC, Canada; ^2^ Département de microbiologie, infectiologie et immunologie, Faculté de médecine, Université de Montréal, Montréal, QC, Canada; ^3^ Département de neurosciences, Faculté de médecine, Université de Montréal, Montréal, QC, Canada; ^4^ Department of Biosciences, COMSATS University, Islamabad, Pakistan; ^5^ Département de médecine, Faculté de médecine, Université de Montréal, Montréal, QC, Canada

**Keywords:** liver, macrophage, acute injury, CCl_4_ (carbon tetra chloride), Kupffer cell (KC), monocyte-derived macrophages, spatial profiling, hepatic stellate cells (HSC)

## Abstract

Macrophages are key regulators of inflammation and repair, but their heterogeneity and multiple roles in the liver are not fully understood. We aimed herein to map the intrahepatic macrophage populations and their function(s) during acute liver injury. We used flow cytometry, gene expression analysis, multiplex-immunofluorescence, 3D-reconstruction, and spatial image analysis to characterize the intrahepatic immune landscape in mice post-CCl_4_-induced acute liver injury during three distinct phases: necroinflammation, and early and late repair. We observed hepatocellular necrosis and a reduction in liver resident lymphocytes during necroinflammation accompanied by the infiltration of circulating myeloid cells and upregulation of inflammatory cytokines. These parameters returned to baseline levels during the repair phase while pro-repair chemokines were upregulated. We identified resident CLEC4F^+^ Kupffer cells (KCs) and infiltrating IBA1^+^CLEC4F^-^ monocyte-derived macrophages (MoMFs) as the main hepatic macrophage populations during this response to injury. While occupying most of the necrotic area, KCs and MoMFs exhibited distinctive kinetics, distribution and morphology at the site of injury. The necroinflammation phase was characterized by low levels of KCs and a remarkable invasion of MoMFs suggesting their potential role in phagoctosing necrotic hepatocytes, while opposite kinetics/distribution were observed during repair. During the early repair phase, yolksac - derived KCs were restored, whereas MoMFs diminished gradually then dissipated during late repair. MoMFs interacted with hepatic stellate cells during the necroinflammatory and early repair phases, potentially modulating their activation state and influencing their fibrogenic and pro-repair functions that are critical for wound healing. Altogether, our study reveals novel and distinct spatial and temporal distribution of KCs and MoMFs and provides insights into their complementary roles during acute liver injury.

## Introduction

Hepatic macrophages are the major liver immune cells. They are a heterogeneous population of cells and have been assigned both beneficial and detrimental roles in human liver disease and experimental models (1). The hepatic macrophage compartment in mice is composed of Kupffer cells (KCs), monocyte-derived macrophages (MoMFs), liver capsular macrophages, and under certain conditions, GATA6^+^ peritoneal macrophages (2–4). Despite the tremendous progress made in determining the origin, transcriptional profile, and function of the different subpopulations of liver macrophages, there are significant gaps in knowledge regarding how these subsets are organized within the tissue and how their spatial distribution may impact cross-talk and division of labor in health and disease (2, 5–9).

KCs are the main subset of hepatic macrophages under normal physiological conditions. They are sessile cells that reside in the sinusoids where they perform crucial tasks during homeostasis, including removal of lipopolysaccharides (LPS), efferocytosis of apoptotic cells, induction of T cell tolerance, and the control of iron and lipid metabolism and bilirubin balance (10). In addition, KCs are sentinel cells and first responders to tissue damage (10). They originate from embryonic yolk sac-derived erythro-myeloid progenitors (YS-KCs) and self-maintain throughout adult life independently of hematopoietic stem cells (7, 11, 12). On the other hand, inflammatory monocytes are absent and MoMFs are present in low numbers in the naïve liver (13). However, during acute liver injury, large numbers of recruited inflammatory monocytes and MoMFs take center stage and carry out various effector functions including clearance of debris and pathogens, activation and resolution of inflammation and extracellular matrix (ECM) remodeling upon injury (1, 6, 8, 10). Notably, infiltrating inflammatory monocytes and MoMFs transition from an inflammatory to a pro-repair phenotype within hours during a typical wound healing response, demonstrating remarkable functional plasticity (6, 8, 14). This conversion occurs at the injury site and depends on IL-4, IL-10, phagocytosis, and neutrophil-derived reactive oxygen species (ROS) (6, 8, 15). Interestingly, KCs and MoMFs, in addition to their differential functionality at various levels of liver disease, exhibit distinct expression of various cell surface markers. In mice, KCs are CD11b^int^, F4/80^hi^, CLEC4F^+^ cells. MoMFs are Ly6C^+^ CX3CR1^+^ and originate from recruited inflammatory monocytes (Ly6C^hi^ CX3CR1^low^) (1, 6, 8, 10). Another important function of liver macrophages is their capacity to activate hepatic stellate cells (HSCs), which enhances their fibrogenic and pro-repair functions (1). However, the role of the various hepatic macrophage subsets in HSC activation is not fully understood.

In this study, we undertook a spatial and temporal characterization of the liver macrophage compartment to better understand how the different macrophage subsets interact during steady state and in response to injury. We used the carbon tetrachloride (CCl_4_) acute injury model in mice as it recapitulates immunological, histological, and pathological features of human toxic liver injury (16). We established the dynamics of the two largest macrophage subpopulations that spatially overlap in the necrotic areas around central veins (CVs) in response to acute hepatic injury: KCs and MoMFs. Despite their proximity, these subsets exhibited major differences regarding their origin, time of necrotic tissue infiltration, position at the injury site, morphology, capacity to replenish the macrophage pool during tissue repair, and colocalization with HSCs. These results add spatial dimension and tissue context to the interplay between MoMFs and KCs and complement the large body of functional and transcriptomics studies that define these major macrophage subsets and their reciprocal interactions.

## Materials and methods

### Mice

Eight- to ten weeks old C57BL/6N male mice were obtained from Charles River Laboratories (Senneville, QC, Canada; strain code 027). Animals were maintained in a specific pathogen–free facility at the Centre de Recherche du Centre hospitalier de l’Université de Montréal (CRCHUM). All animal experiments were approved by the Institutional Animal Care and Use Committee (Protocol IP18035NSs). Mice received a single intraperitoneal injection of CCl_4_ (Sigma-Aldrich, Oakville, ON, Canada) at 1 ml/kg resuspended in corn oil (Sigma-Aldrich). Control mice received corn oil only. Mice were terminally euthanized with 400 mg/kg Euthanyl (sodium pentobarbital; CDMV, St-Hyacinthe, QC, Canada) at 0, 12, 24, 48, 72, 96, and 168 h post injection.

### Histology

Hematoxylin and eosin (H&E), Ki67 and IBA1 immunohistochemistry (IHC) stainings were performed by the molecular pathology core platform of the CRCHUM using the Shandon multiprogram robotic slide stainer on 4 μm formalin-fixed paraffin-embedded (FFPE) hepatic whole tissue sections. Images were acquired with the whole slide scanner Olympus BX61VS. Quantification of the necrotic area was performed with the VIS software (Version 2018.4, Visiopharm, Hørsholm, Denmark) using protocols that automatically detected the tissue and manual delineation of the necrotic area by visual inspection. References for antibodies and H&E reagents are provided in **Tables S1, S2** in the Supplementary Material.

### Alanine aminotransferase activity assay

ALT test was performed on murine plasma by the clinical laboratory at the Centre hospitalier de l’Université de Montréal. Briefly, mice were bled by cardiac puncture and the blood was anticoagulated using 10 μL of 10% K_2_EDTA in H_2_O per 1 mL of blood. The blood was centrifuged at 13000 rpm for 5 minutes at room temperature, and the upper phase containing the plasma was collected and stored at -80°C until ready for ALT activity measurement.

### Multiplex immunofluorescence

Multiplex immunofluorescence (mIF) for CLEC4F, IBA1, αSMA, GATA-6, and Desmin was performed on 4 μm FFPE tissue sections as previously described (17). mIF for IBA1, CLEC4F, and MARCO was done on frozen 5μm OCT-embedded and fixed liver sections. Briefly, sections were kept at -80°C before usage. Sections were aired 5 minutes at room temperature (RT) and then immersed in washing buffer for 10 minutes for OCT removal. Next, sections were transferred to another jar containing antigen retrieval buffer and subjected to high pressure for 5 minutes inside an electric pressure cooker. The pressure was released, the jar was taken out and sections were left for another 20 minutes inside the antigen retrieval buffer to cool down. Afterwards, sections were washed and then incubated with glycine solution for 10 minutes at RT, washed two times and then incubated 30 minutes with blocking solution at RT. Next, sections were washed and incubated overnight with the cocktail of primary antibodies in blocking solution at 4°C inside a humidity chamber, to be washed again and incubated with the cocktail of secondary antibodies during 30 minutes at RT in the dark. Finally, washed sections were mounted in Slowfade Gold mounting media plus DAPI and stored in the dark at 4°C up to the acquisition time. mIF for IBA1, CLEC4F, CCR2 and CX3CR1 was performed on 5μm frozen sections. Briefly, sections were kept at -80°C before usage. They were aired 5 minutes at room temperature (RT) and then immersed in tissufix for 10 minutes at RT for mild fixation. Next, sections were washed and subjected to the same protocol as described above for OCT sections. All antibodies, solutions, and reagents used are listed in **Supplementary Table S1**. Whole tissue images were acquired using the whole slide scanner Olympus BX61VS. We performed staining on serial sections when we could not multiplex the primary antibodies of different markers because they were raised in the same species or when the purpose was to align images originated using different techniques (e.g., IHC and H&E).

To mitigate any autofluorescence issues, we tried to use IF channels having the lowest autofluorescence in the liver and/or that have little spill over. In addition, we performed quantification using the VIS software that allows the establishment of thresholds of positivity for every individual channel, with pixel intensity values set well above the background signal and visual artifacts removed from the regions to be analyzed. Non-specific binding of primary antibodies was ruled out using specific staining pattern for each Ab corresponding to the tissue or cellular location of the marker to be assessed (i.e. nuclear, cytoplasmic, around CV area, etc.). The specificity of the stain was confirmed using other antibodies raised in the same species as negative controls.

### RNA isolation and RT-PCR analysis

Total RNA was isolated from murine hepatic tissue using RNeasy Mini Kit (Qiagen). cDNA was generated from 2 µg of total RNA using the Transcriptor Universal cDNA Master Mix (Roche Life Science). cDNA was amplified using the LightCycler^®^ 480 SYBR Green I Master (Roche) in the LightCycler 480 instrument (Roche). All the previous procedures were performed according to the manufacturer’s protocols. mRNA expression was normalized to the expression of the housekeeping gene 28s and was determined using the 2-^ΔΔCt^ method. Primer sequences are listed in **Supplementary Table S2**.

### Image analysis

Image analysis was performed using the image analysis software VIS. The Author™ module was used to design the following protocols: 1) Automated identification and quantification of tissue area; 2) Identification and quantification of IBA1^+^CLEC4F^-^ area; 3) Identification and quantification of IBA1^+^CLEC4F^+^ area; 4) Quantification of αSMA^+^ area; 5) Quantification of the colocalization between αSMA^+^ cells and CLEC4F^+^ cells versus IBA1^+^CLEC4F^-^ cells; 6) Measurement of the distance from nuclei to central vein. The Tissuealign module of VIS was used for automated alignment of images from serial sections. The tissue heatmap function of VIS was used for generating heatmaps for CLEC4F^+^ KCs and IBA1^+^CLEC4F^-^ macrophages. The detection of nuclei was done using the protocol 10169 – Nuclei Detection, AI (Fluorescence) from Visiopharm.

### Isolation of intrahepatic leukocytes (IHLs)

IHLs were isolated from hepatic tissue in a two-step process: first by mechanical dissociation and next by enzymatic digestion. First, the liver was minced using a scalpel. The dissociated tissue was suspended in cold RPMI media supplemented with 10% FBS plus benzonase (0.2 U/mL) and collagenase D (0.1 mg/mL), and then incubated at 37°C for 30 minutes with rotation. Upon incubation, the tissue was passed through a 70 μm cell strainer for further mechanical dissociation. The tissue suspension was washed with cold RPMI/FBS, layered on a percoll discontinuous gradient (80% bottom, 40% top), and centrifuged at 1500 rpm for 25 minutes. The IHLs were recovered from the interphase between the 2 percoll solutions and resuspended in fresh media. The references of all reagents used in this protocol are listed in **Supplementary Table S2**.

### Flow cytometry

For detection of surface markers, freshly isolated IHLs were washed with FACS buffer (PBS, 1% FBS, 0.02% Sodium Azide) and then transferred to a 96 well plate where they were incubated with a cocktail of fluorescently conjugated antibodies plus the viability dye aqua vivid (Thermofisher) and 10% mouse serum for 30 minutes at 4°C in the dark. IHLs were then washed two times with FACS buffer and then fixed in 1% formaldehyde in PBS and kept at 4°C in the dark up to acquisition time. In cases where intracellular staining was required, after labelling cell surface markers, IHLs were incubated with permeabilization/fixation FOXP3 buffer (eBioscience) for 30 minutes at 4°C in the dark. Next, IHLs were washed and incubated with the cocktail of antibodies for intracellular targets for 30 minutes at 4°C in the dark. Finally, the samples were washed and fixed and kept at 4°C in the dark up to acquisition time. To detect spontaneous cytokine production, IHLs were incubated with 10 µg/ml of Brefeldin A (Sigma-Aldrich) and 6 µg/ml of monensin sodium salt (Sigma-Aldrich) for 6 h prior to staining.

Samples were acquired on a BD LSRFortessa™ Cytometer equipped with violet (405 nm), blue (488 nm), yellow-green (561 nm) and red (633 nm) lasers and FACSDiva version 8.0.1 (BD Biosciences, San Diego, CA). Analysis was performed using FlowJo version 10.4 for Mac (BD Biosciences, Ashland, OR) and FlowSOM.

### Three-dimensional reconstruction

Sixteen μm sections from frozen liver OCT embedded sections from 48 h CCl_4_ treated mice were imaged using αSMA, CLEC4F, and IBA1 specific antibodies according to multiplex immunofluorescence protocol described above. Nuclei were counterstained using DAPI. The images or z-stacks were acquired using 40× objective, ApoTome 2, Zeiss. Three-dimensional (3D) reconstruction was done using Imaris 8.1.2 software (Bitplane, Zurich, Switzerland).

### Statistical analysis

Statistical analysis was performed with GraphPad Prism 9 software. Data are presented as mean ± SEM. The number of samples for each experiment and the number of replicate experiments are indicated in the figure legends. Statistical significance between two groups was determined by Mann-Whitney Test. When comparing more than two groups, analysis of variance (ANOVA) followed by Dunn’s Multiple Comparison Test was performed. A *P* value < 0.05 was considered statistically significant.

## Results

### Distinct necroinflammatory and tissue repair phases are observed during acute liver injury

**Figure 1:**
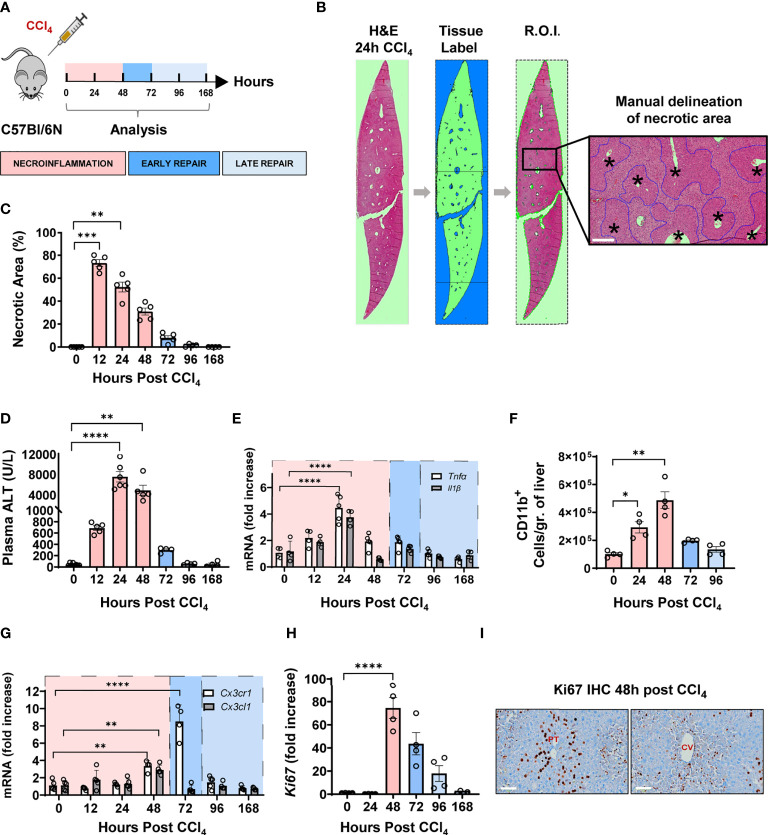
CCl_4_-induced acute liver injury is characterized by a necroinflammatory phase followed, at 48 h post injury, by an early tissue repair phase and then a late one from 72 hours onward. **(A)** Schematics of the experimental design delineating the phases of the wound healing response to one intraperitoneal injection of CCl_4_ at 1μL/g of body weight. **(B)** Representative H&E image of one whole liver section covering at least the total surface area of a transverse section of one entire lobe per mouse, at 24 h following CCl_4_ injection. Necrotic area was delineated manually around CV as outlined in blue, scale bar=200 μm, * indicates CV. **(C)** Percentage of necrotic area within the total tissue area. **(D)** Plasma ALT levels. **(E)** Relative gene expression levels of *Tnfα* and *Il1β* determined by qPCR on bulk liver tissue. The mRNA expression data represent fold increase relative to 0 h controls and was normalized to 28s. **(F)** Kinetics of recruitment of intrahepatic myeloid cells as determined by flow cytometry. **(G)** Relative gene expression levels of *Cx3cr1* and *Cx3cl1* determined by qPCR on bulk liver. **(H)** Relative gene expression levels of *Ki67* determined by qPCR on bulk liver. **(I)** Representative Ki67 immunohistochemistry images from liver sections of 48 h CCl_4_-treated mouse showing Ki67 staining around portal tracts (PT) on the left and CVs on the right, scale bar 75 μm. N=4-5 mice per group. Data are shown as mean ± SEM. Statistical analysis was performed using one-way ANOVA followed by Dunn’s Multiple Comparison Test. *P < 0.05, **P < 0.01, ***P < 0.001, ****P < 0.0001.

We sought to understand the wound healing response to acute liver injury in mice using the CCl_4_ model, which is characterized by central vein (CV) injury and a self-limited immune response leading to total healing (16). Herein, 8-10 weeks old C57BL/6N male mice received a single intraperitoneal injection of CCl_4_ (1 µl/g of body weight), and livers and plasma were collected and analyzed at 12, 24, 48, 72, 96 and 168 hours post-injection (**Figure 1A**). Importantly, all imaging labelling, analysis and quantification are done on whole tissue sections (**Figure 1B**) with quantification undertaken using automatic protocols with predefined thresholds for signal positivity (17). H&E staining of liver sections demonstrated the hallmark characteristics of CV-associated injury and extensive immune cell infiltration in the necrotic tissue (**Figure 1B**). We assessed tissue damage by quantifying the percentage of necrotic area and ALT plasma levels. We observed that hepatocellular necrosis was ongoing at 12 h, peaked at 24 h, and persisted up to 48 h post-CCl_4_. At 72 h, both the necrotic area and ALT levels returned to normal suggesting that the period between 48 and 72 h was critical for the transition to tissue repair (**Figures 1C, D**). The hepatic expression of genes encoding the pro-inflammatory cytokines TNFα and IL-1β closely mirrored the kinetics of plasma ALT, with peak expression at 24 h and return to baseline at 72 h (**Figure 1E**). In addition, flow cytometric analysis showed a considerable influx of CD11b^+^ myeloid cells into the liver between 24 – 48 h, returning to baseline levels at 72 h (**Figure 1F**). Furthermore, the hepatic gene expression of the pro-resolving chemokine receptor-ligand pair CX3CR1-CX3CL1 was upregulated at 48-72 h post-CCl_4_ marking the transition to tissue repair (**Figure 1G**). Interestingly, the proliferation marker Ki67 was upregulated, both at the mRNA and protein levels, at 48 h and to a lesser extent at 72 h post-injury (**Figures 1H, I** and **Supplementary Figure S1**). Immunohistochemical Ki67 staining showed massive and compartmentalized proliferation of parenchymal (hepatocytes with large round nuclei) and non-parenchymal cells (small irregularly shaped nuclei) around portal tracts and CVs, respectively, at 48 h post-CCl_4_ (**Figure 1I**). Altogether, these data allowed us to delineate three distinct phases of wound repair during acute liver injury (**Figure 1A**). First, the necroinflammatory phase, characterized by tissue damage, increased levels of inflammatory cytokines, and immune cell infiltration, spans from 0 to 48 h post-CCl_4_. Second, the early phase of tissue repair from 48 to 72 h post-CCl_4_, characterized by receding inflammation with return to baseline levels of inflammatory cytokines and tissue damage markers, and concomitant upregulation of pro-resolving genes and proliferation of tissue cells. Third, the late repair phase between 72 to 168 h post-CCl_4_ where inflammation and tissue damage indicators return to homeostatic levels.

### CCl_4_-mediated acute liver injury caused partial and temporary depletion of hepatic resident immune populations and massive influx of circulating myeloid cells

**Figure 2:**
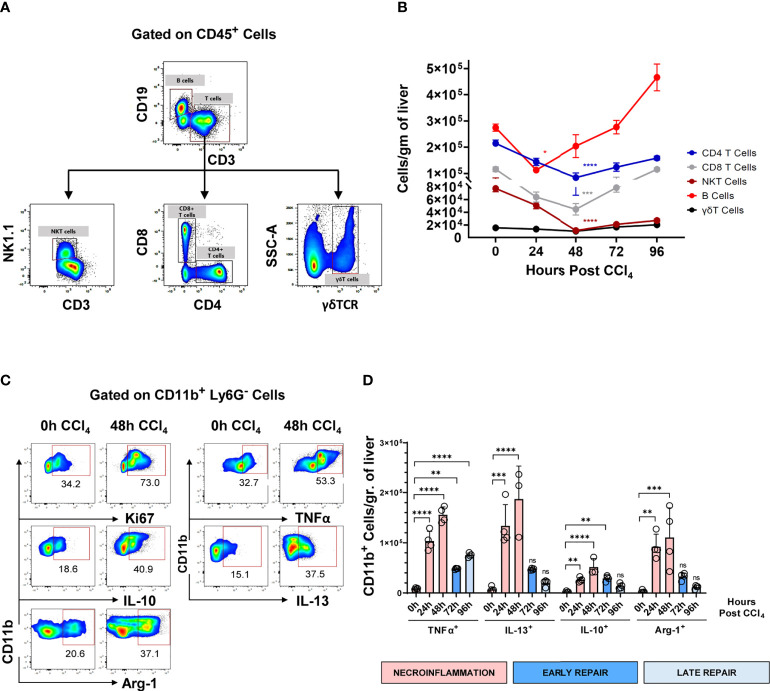
Hepatic resident lymphocytes are partially depleted during the necroinflammatory phase and recover during the tissue repair phase of CCl_4_-mediated acute liver injury, while intrahepatic myeloid cells respond by inflammatory mediators’ release. **(A)** Gating strategy for flowcytometric analysis of intrahepatic lymphocytes, gated on live CD45+ lymphocytes. **(B)** Kinetics of the main hepatic resident lymphocyte populations following one single intraperitoneal injection of CCl_4_ at 1μL/g of body weight. **(C)** Representative pseudocolor plots of analysis spontaneous production of different immune mediators by intrahepatic myeloid cells treated for 6h with BFA and monensin, presented as frequencies of CD11b^+^ Ly6G^-^ cells. **(D)** Expression of TNFα, IL-10, IL-13, and Arg-1 normalized to the number of CD11b^+^ myeloid cells/gr of liver during the different phases of the response to CCl_4_-mediated acute liver injury. N=4-5 mice per group. Data are shown as mean ± SEM. Statistical analysis was performed using one-way ANOVA followed by Dunn’s Multiple Comparison Test. *P < 0.05, **P < 0.01, ***P < 0.001, ****P < 0.0001. ns, not significant.

Next, we characterized the intrahepatic leucocytes during the three different phases of acute injury using high-resolution flow cytometry. We observed that the major resident lymphocyte populations including T cells, B cells, and NKT cells, were significantly reduced during necroinflammation and only recovered during tissue repair (**Figures 2A, B**). Conversely, as demonstrated above, infiltrating CD11b^+^ myeloid cells significantly increased during necroinflammation (**Figure 1F**). These myeloid cells upregulated Ki67 expression suggesting that they underwent *in-situ* proliferation (**Figure 2C**). In addition, we observed that CCl_4_-mediated injury induced expression of pro-inflammatory cytokines like TNFα, the pro-fibrogenic cytokine IL-13, and the anti-inflammatory mediators IL-10 and Arg-1 by hepatic myeloid cells, suggesting that they may be functionally implicated in both necroinflammation and tissue repair (**Figures 2C, D**).

**Figure 3:**
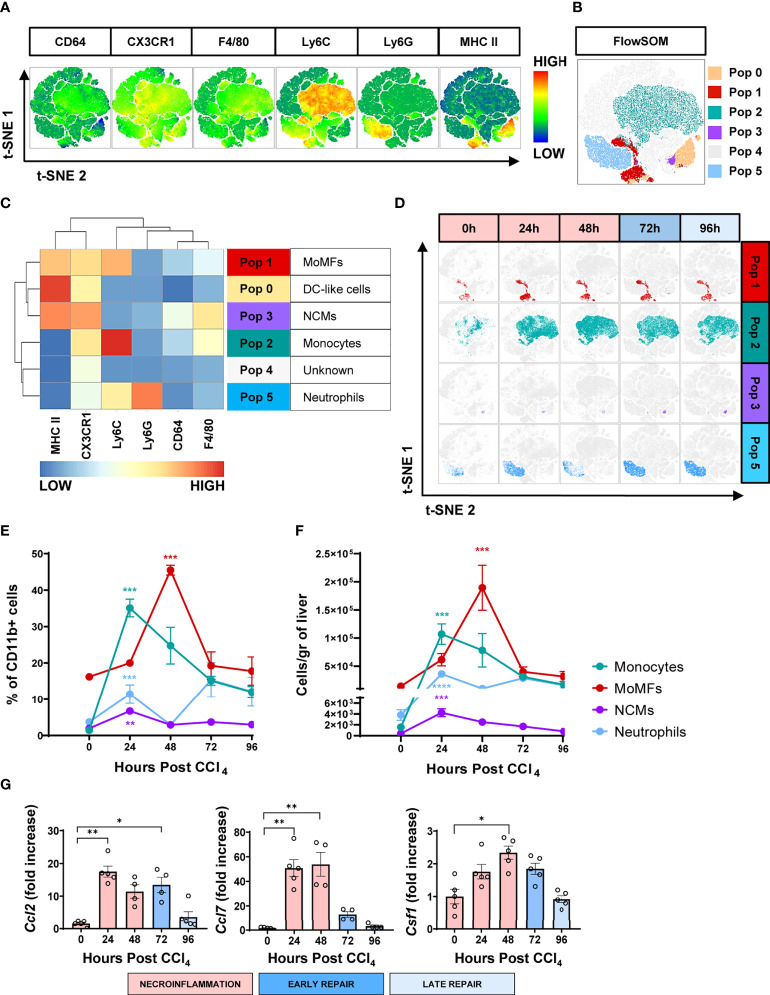
The wound healing response to acute liver injury is associated with an influx of circulating myeloid cells. **(A)** t-SNE plots of total myeloid CD11b^+^ cells showing pooled expression of myeloid-associated markers from all mice at all time points. **(B)** t-SNE projection of myeloid cell populations identified by FlowSOM. **(C)** Heatmap showing relative marker expression associated with the different myeloid populations identified by FlowSOM and their proposed identity. **(D)** Representative t-SNE plots showing the kinetics of MoMFs, inflammatory monocytes, NCMs and neutrophils in response to acute liver injury. **(E)** Frequencies of the different myeloid cell populations as determined by manual gating. **(F)** Total number of myeloid cell populations per gram of liver as determined by manual gating. **(G)** Relative gene expression of *Ccl2*, *Ccl7* and *Ccsf1* determined by qPCR on bulk liver tissue. The mRNA expression data represent fold increase relative to 0 h controls and was normalized to 28s. N=4 mice per group. Data are shown as mean ± SEM. Statistical analysis was performed using one-way ANOVA followed by Dunn’s Multiple Comparison Test. *P < 0.05, **P < 0.01, ***P < 0.001, ****P < 0.0001.

For unbiased identification of the myeloid subpopulations induced upon acute injury, we applied t-distributed stochastic neighbor embedding (t-SNE) analysis and FlowSOM on flow cytometry data for several well-established myeloid markers (18). These analyses were undertaken at 0, 24 h, 48 h, 72 h and 96 h post-CCl_4_-injury. The t-SNE heatmap density plots of the pooled data for each marker are presented in **Figure 3A**. Unsupervised clustering generated by FlowSOM identified six CD11b^+^ populations (**Figures 3B-D**). Population 0 may represent a subset of hepatic dendritic cells because they are F4/80^-^ Ly6C^-^ CX3CR1^+^ MHCII^hi^, but definitive confirmation requires the inclusion of the CD11c marker that is absent in this analysis (11, 19). Population 1 was classified as MoMFs as they expressed Ly6C and CX3CR1, and moderate levels of F4/80 and MHC II (8, 10). Population 2 was classified as inflammatory monocytes due to its high expression of Ly6C, low to negative expression of CX3CR1, and no expression of the granulocytic marker Ly6G (6, 10, 15, 20, 21). Population 3 was classified as non-classical monocytes (NCMs) since they were Ly6C^-^ Ly6G^-^ CX3CR1^hi^ MHCII^+^ F4/80^+^ (22, 23). Population 4 could not be classified with the markers included in this analysis. Population 5 expressed Ly6G and intermediate levels of Ly6C and was classified as neutrophils (10, 24). The kinetics of the different myeloid subpopulations are shown in representative t-SNE plots in **Figure 3D**. We also validated the FlowSOM population identity for the four phagocytic myeloid populations identified above using manual gating (**Supplementary Figure S2A**). Representative flow cytometry plots of the kinetics of myeloid populations are presented in **Supplementary Figure S2B**. Surprisingly, t-SNE FlowSOM analysis did not reveal a population with a phenotype consistent with KCs. The two predominant populations expressing high levels of the macrophage marker F4/80 (populations 2 and 3) also co-expressed markers inconsistent with the KC phenotype (Ly6C and CX3CR1). It is important to note here that we also tried gating manually on KCs, using phenotypic markers set previously as CD64^+^ MHCII^+^ Ly6C^low/int^ CD11b^int^ F4/80^high^ cells (10). However, in our experiments CD11b^+^ MHC II^+^ F4/80^+^ CD64^+^ were also CX3CR1^+^ thus confirming that they are not KCs (**Supplementary Figure S3**). Altogether, we identified four different myeloid cell populations implicated in acute liver injury, which is consistent with MoMFs, inflammatory monocytes, NCMs and neutrophils.

### Kinetics of myeloid populations at the liver injury site reveal temporally distinct waves of inflammatory monocytes, NCMs, neutrophils and MoMFs

Next, we examined the infiltration kinetics of the four main myeloid populations identified. The numbers of inflammatory monocytes and neutrophils were very low in the hepatic tissue at the steady state (**Figures 3E, F**). This situation dramatically changed during the first half of the necroinflammatory phase (12 to 24 h post-CCl_4_), when inflammatory monocytes and a first wave of neutrophils extensively infiltrated the hepatic tissue (**Figures 3E, F**), consistent with previous reports in the acetaminophen (APAP)-induced acute hepatic injury model (10, 20). Interestingly, NCMs, normally associated with anti-inflammatory and pro-repair effector functions, also increased during this period, suggesting that they may be involved in preventing collateral damage associated with the inflammatory reaction (22, 23, 25). The second half of the necroinflammatory stage (24 to 48 h post-CCl_4_) was characterized by the simultaneous decline of the above-mentioned inflammatory populations and the rapid increase of MoMFs, outnumbering all other phagocytic subsets and prevailing during the early repair phase (**Figures 3E, F**). We also observed increased expression of the genes encoding monocyte/macrophage chemokines *Ccl2* and *Ccl7* and the growth factor *Csf1* during necroinflammation and early repair, suggesting their involvement in the observed monocyte/macrophage recruitment and expansion (**Figure 3G**), as previously reported (7, 12, 26, 27). Finally, two waves of neutrophils infiltrated the hepatic tissue at the peak of necroinflammation and repair stages at 24 h and 72 h, respectively (**Figures 3E, F**). In summary, whereas tissue-resident immune populations decreased during necroinflammation, circulating myeloid cells infiltrated the liver in an orchestrated fashion and their presence was temporally associated with the restoration of tissue homeostasis.

### Acute liver injury induces changes in the composition, density, and spatial distribution pattern of the hepatic macrophages

To understand the functional and spatial implications of the different macrophage subsets, we proceeded to map the macrophage populations *in-situ* in the CCl_4_-injured liver tissue. Even though characterization of macrophages in acute liver injury has previously been described, the spatial and temporal distribution of the two major subpopulations, KCs and MoMFs and their interrelation to one another during the wound healing response are still unknown. In addition, previous studies on liver macrophages have relied on F4/80 for tissue visualization, however, this marker does not discriminate between different macrophage subpopulations (10, 20, 28).

**Figure 4:**
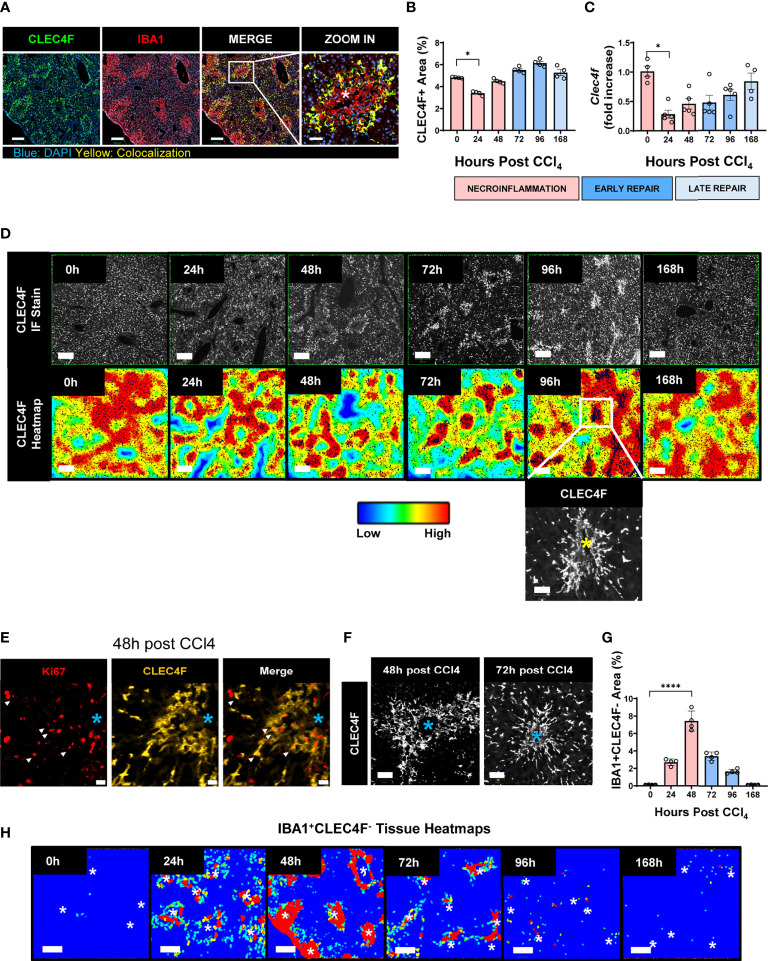
KCs and MoMFs exhibit different distribution patterns and kinetics during acute liver injury whereby early transient depletion of CLEC4F^+^ KCs is followed by recruitment of IBA1^+^CLEC4F^-^ macrophages then return to baseline conditions during repair. **(A)** mIF representative images of liver tissue at 48 h post-CCl_4_. From left to right, CLEC4F (green), IBA1 (red), merge/colocalization (yellow). Nuclei are stained with DAPI (blue) scale bar =250 μm; the inset showing merge image at high magnification, scale bar =60 μm, *indicates CV. **(B)** Density of CLEC4F^+^ KCs macrophages as assessed by IF and expressed as % of total tissue area. **(C)** Quantification of the *Clec4f* by qPCR. The mRNA expression data represent fold increase relative to 0 h controls and was normalized to 28s. N=4-5 mice per group **(D)** Representative CLEC4F immunofluorescence images and their respective tissue heatmaps, indicating density per pre-defined area unit (color assigned according to a density grade), scale bar =250 μm. Inset showing high magnification of original CLEC4F staining, scale bar =50 μm. **(E)** IF representative images of Ki67 (red), CLEC4F (yellow) at 48 h, scale bar =20 μm. **(F)** High magnification of CLEC4F IF at 48 h (left) and 72 h (right) post-CCl_4_, scale bar 70 μm. **(G)** Density of IBA1^+^CLEC4F^-^ macrophages as assessed by IF and expressed as % of total tissue area. **(H)** Representative tissue heatmaps of IBA1^+^CLEC4F^-^ macrophages, scale bar =250 μm. High density areas displayed in red and low density displayed in blue, intermediate values displayed according to the color scale in the figure. N=4-5 mice per group. Data are shown as mean ± SEM. Statistical analysis was performed using one-way ANOVA followed by Dunn’s Multiple Comparison Test. *P < 0.05, ****P < 0.0001.

We assessed liver macrophages using the KC specific marker, uniquely expressed by KCs regardless of their origin (YS-KCs *vs.* bone marrow (BM)-KCs) (14), the C-Type Lectin Domain Family 4 Member F (CLEC4F), and the macrophage activation marker, the ionized calcium-binding adapter molecule 1 (IBA1) (26, 27, 29). IBA1/CLEC4F multiplex immunofluorescence (mIF) of liver tissue revealed two distinct subpopulations of macrophages infiltrating the necrotic areas around CVs during acute liver injury: IBA1^+^CLEC4F^+^ KCs (CLEC4F^+^ KCs) and IBA1^+^CLEC4F^-^ macrophages (**Figure 4A**). We were capable of simultaneously detecting CLEC4F^+^ KCs and IBA^+^CLEC4F^-^ macrophages in the same sections demonstrating their distinct spatial distribution at the injury site (**Supplementary Figure S4**), whereby IBA^+^CLEC4F^-^ macrophages were closer to CVs and surrounded by CLEC4F^+^ KCs. These two hepatic macrophage populations were still detectable around injured CVs during chronic liver injury and fibrosis, as shown by mIF staining of IBA1 and CLEC4F in fibrotic liver sections at 12 weeks post-CCl_4_ (**Supplementary Figure S5**). Quantification of CLEC4F^+^ area showed that KCs were partially depleted during necroinflammation and recovered during repair (**Figure 4B**) (5, 10). In accordance, our qPCR data demonstrated decreased *Clec4f* gene expression further confirming KC depletion at necroinflammation and suggesting death of KCs at this phase given the massive tissue damage revealed at 24 h by ALT levels (**Figure 4C**).

We further generated tissue heatmaps to provide an overview of the tissue distribution pattern and evolution of these subsets during acute liver injury. Tissue heatmaps of CLEC4F^+^ KCs showed shrinkage of densely populated red areas during necroinflammation, consistent with KC partial depletion (**Figure 4D**, 24 h). Conversely, during repair, starting at 48 h and extending to 96 h, CLEC4F^+^ KCs proliferated around healing CVs, as shown by the increasing red spot areas (**Figure 4D**, 48 h, 72 h and 96 h), and their quantification (**Figure 4B**). In addition to the upregulated Ki67 expression on these KCs, as shown by the multitude of Ki67^+^ nuclei in the area occupied by CLEC4F^+^ cells (**Figure 4E**). Indeed, another support of a proliferative phenotype of CLEC4F^+^ cells at repair, is that colocalization of CLEC4F and Ki67 at 48 h around CVs preceded the formation of highly packed clusters of KCs at 72h and 96h post -CCl_4_ (**Figures 4E, F**). These aggregates of CV-associated CLEC4F^+^ KCs dissipated during the late repair phase (96 h-168 h). Our tissue mapping also demonstrated that KCs returned to their normal tissue density and distribution by 168 h post-CCl_4_ (**Figure 4D**, 168 h). It is important to note here that we labelled KCs with the specific marker, CLEC4F, and we essentially observed the same staining pattern as observed in previous studies using F4/80 labelling where KCs are enriched around portal tracts in the steady state (30), but their distribution changes during necroinflammation and tissue repair as shown in **Figures 4A, D**.

In contrast, IBA1^+^CLEC4F^-^ macrophages were absent in the uninjured liver and were recruited in large numbers upon injury (**Figure 4G**). Tissue heatmaps revealed that infiltrating IBA1^+^CLEC4F^-^ macrophages were restricted to the necrotic areas around CVs during necroinflammation and early repair, and disappeared by the late repair phase (**Figures 4G, H**). In summary, resident CLEC4F^+^ KCs and infiltrating IBA1^+^CLEC4F^-^ macrophages exhibited different kinetics and distinctive distribution patterns supporting the notion of unique roles and division of labor between these two subsets of macrophages. Our staining strategy revealed the distinct distribution patterns of the two hepatic macrophage populations at the injury site.

### The necrotic area during acute liver injury is characterized by distinct temporal distribution and microanatomical localization of CLEC4F^+^ KCs and IBA1^+^CLEC4F^-^ macrophages

**Figure 5:**
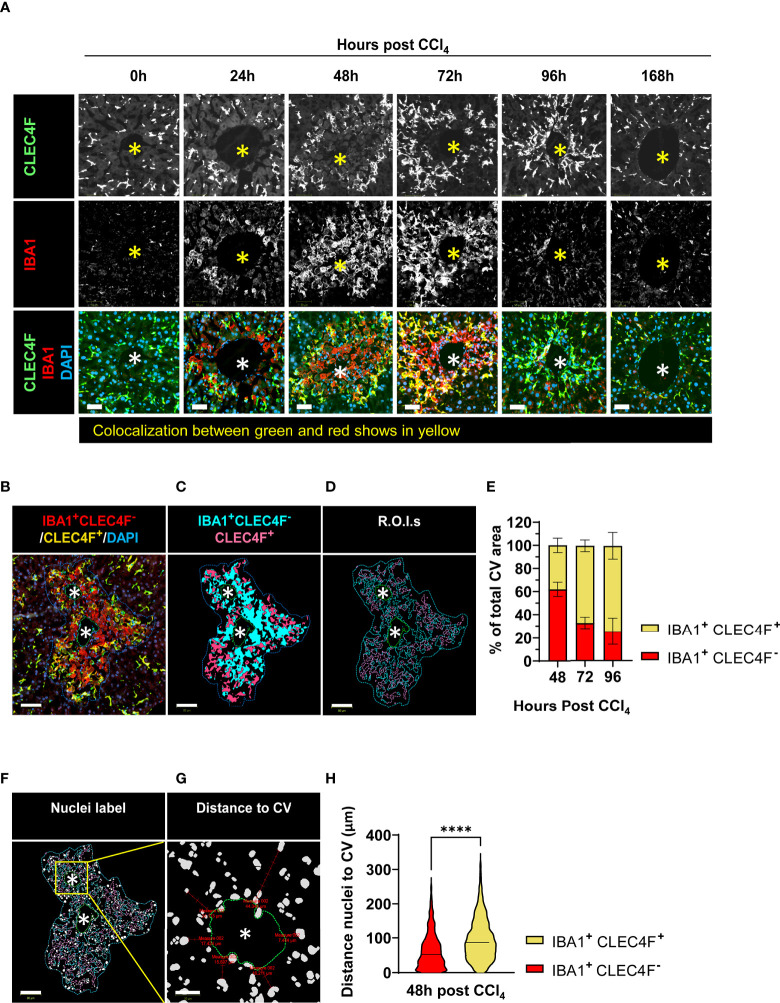
CLEC4F^+^ KCs and IBA1^+^CLEC4F^-^ macrophages are in close contact in the necrotic area but exhibit different kinetics, microanatomical location, and morphology. **(A)** Representative mIF images of CLEC4F (green), IBA1 (red) and DAPI (blue) around injured CVs. Top, middle, and bottom rows show CLEC4F single channel (grayscale), IBA1 single channel (grayscale) and merge (CLEC4F in green, IBA1 in red and DAPI in blue), respectively, scale bar =50 μm, * indicates CV. **(B)** Representative image of CV-associated macrophages at 48 h post-CCl_4_ with CLEC4F^+^ KCs in yellow and IBA1^+^CLEC4F^-^ macrophages in red, scale bar =80 μm. **(C)** Digitally generated labels for CLEC4F^+^ KCs (pink) and IBA1^+^CLEC4F^-^ macrophages (cyan). **(D)** Automated outlining of R.O.I. with dotted lines for the necrotic area (blue), CLEC4F^+^ KCs (pink), IBA1^+^CLEC4F^-^ macrophages (cyan) and CV (green). **(E)** Automated measurement of CLEC4F^+^ KCs (pink cells in D) and IBA1^+^CLEC4F^-^ macrophages (cyan cells in D) to calculate the percentage of necrotic area occupied by each of these subpopulations. **(F)** Automated detection of nuclei inside the R.O.I.s. **(G)** Automated measurement of shortest distance from each nucleus to CV. **(H)** Quantification of the distance to the CV from IBA1^+^ CLEC4F^-^ nuclei vs. IBA1^+^ CLEC4F^+^ cells nuclei. N=4 mice per group. Data are shown as mean ± SEM, n>4000 nuclei. Statistical analysis was performed using Mann–Whitney U test. ****P < 0.0001.

Given that most of the dynamic changes take place around necrotic CVs, we further characterized CLEC4F^+^ KCs and IBA1^+^CLEC4F^-^ macrophages in this zone. At the steady state, areas around non-injured CVs were populated by resident CLEC4F^+^ KCs exhibiting weak to negative IBA1 expression (**Figure 5A**, 0 h) while IBA1^+^CLEC4F^-^ macrophages were absent in the naïve liver. These CLEC4F^+^ KCs exhibited the typical sinusoidal location, elongated processes, and the scattered distribution of KCs. During necroinflammation (at 24 h), CLEC4F^+^ KCs were located primarily in the periphery of the necrotic area, and some of them displayed yellow labeling probably due to upregulation of IBA1 expression. Concomitantly, IBA1^+^CLEC4F^-^ macrophages appeared in this region (**Figure 5A**, 24 h). These two subsets of macrophages exhibited distinct cell morphologies: stellar-shaped in the case of CLEC4F^+^ KCs, and globular in the case of IBA1^+^CLEC4F^-^ macrophages (**Figure 5A**, 24 h). At 48 h, CLEC4F^+^ KCs (yellow cells) formed ring-shaped structures in the periphery of clusters of IBA1^+^CLEC4F^-^ macrophages (red cells) that filled the inner necrotic area closer to CVs (**Figure 5A**, 48 h). During early repair, the population of IBA1^+^CLE4F^-^ macrophages was markedly reduced to finally disappear by 96 h to 168 h post-CCl_4_. Conversely, dense aggregates of CLEC4F^+^ KCs moved closer to the CVs between 72 and 96 h and then dispersed by 168 h post-CCl_4_ (**Figure 5A**, 96 h and 168 h).

Next, we measured the relative area occupied by CLEC4F^+^ KCs and IBA1^+^CLEC4F^-^ macrophages in the necrotic area (around CVs) between 48 to 96 h post-CCl_4_ (**Figures 5B-E**). Our data confirm that IBA1^+^CLEC4F^-^ macrophages predominate at the necrotic site during the transition from necroinflammation to tissue repair (48 h post-CCl_4_) and declined during late repair (72 - 96 h post-CCl_4_) (**Figures 5B-E**). We sought to evaluate the microanatomical localization of these macrophages relative to CV structures, at 48 h post-CCl_4_, the time point where both subsets are mostly present. We measured the shortest distance from macrophage nuclei to the CV (>4000 nuclei) and found that IBA1^+^CLEC4F^-^ macrophages (red) were closer to CVs than CLEC4F^+^ KCs (yellow) at 48 h post-CCl_4_ (**Figures 5F-H**) suggesting that the two populations occupy different microanatomical locations. To further define cell-cell interactions, we performed three-dimensional (3D) reconstruction and found that stellar-shaped CLEC4F^+^ KCs were in intimate contact with globular IBA1^+^CLEC4F^-^ macrophages at 48 h post-CCl_4_, demonstrating direct contact and suggesting possible interdependence (Video 1, green (KCs) and yellow (MoMFs) cells). Moreover, globularly shaped IBA1^+^ macrophages infiltrated the necrotic/inflammatory area around CV at 24 h post-CCl_4_ (**Supplementary Figure S6**, red arrowheads). Interestingly, hepatocytes with big and round nuclei are visible around CVs at 0 and 24 h (**Supplementary Figure S6**, white arrowheads) but absent at 48 h post CCl_4_. At this time, enucleated hepatocytes around CVs show particulate material inside, indicating that they are necrotic (**Supplementary Figure S6**, inset, yellow arrowheads). Furthermore, digital alignment of serial H&E and IBA1 IHC images revealed that IBA1^+^ macrophages spatially overlapped with necrotic hepatocytes suggesting that these macrophages may be involved in dismantling the nuclei and phagocytosing dead hepatocytes (**Supplementary Figure S6**, red arrowheads, 48 h). In summary, resident CLEC4F^+^ KCs and infiltrating IBA1^+^CLEC4F^-^ macrophages occupied most of the necrotic area around CVs, suggesting that they may be critical players in the wound healing response to acute injury. These two hepatic macrophage subpopulations differed in their kinetics, phenotype, cell morphology and microanatomical tissue location.

### CV-associated IBA1^+^CLEC4F^-^ macrophages exhibit characteristics of MoMFs

**Figure 6:**
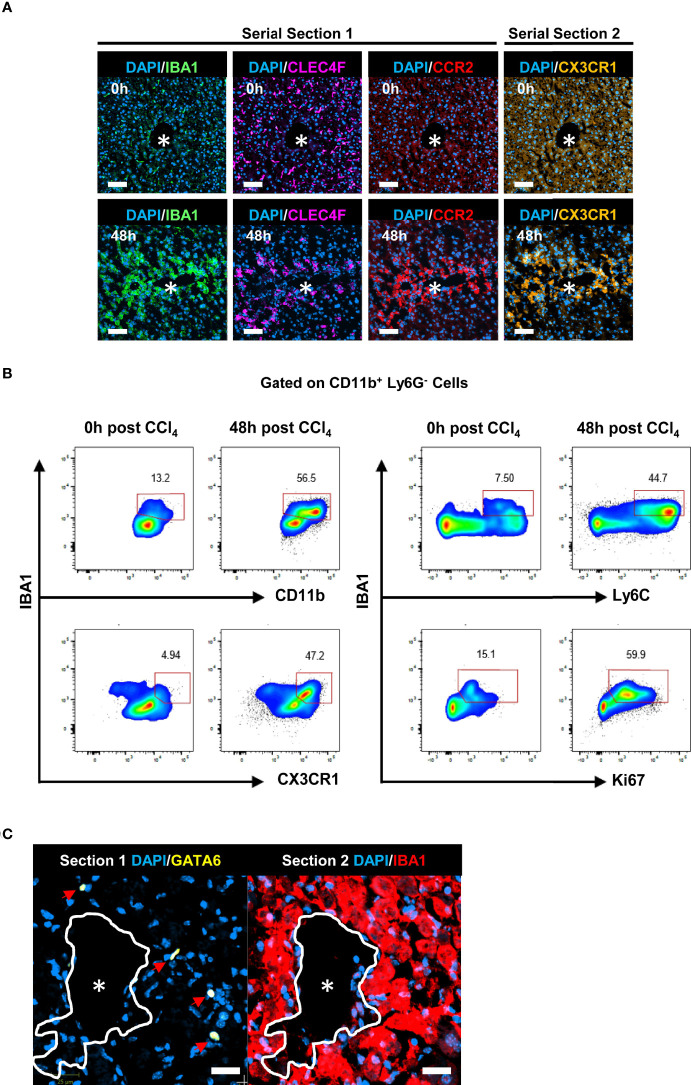
IBA1^+^CLEC4F^-^ macrophages exhibit phenotypic markers of MoMFs. **(A)** Representative mIF images of IBA1 (green), CLEC4F (magenta) and the monocytic markers CCR2 (red), CX3CR1 (orange) and DAPI (blue) around CVs at 0 h vs. 48 h post-CCl_4_, scale bar= 75 μm. IBA1, CLEC4F and CCR2 were multiplexed on the same section (section 1), CX3CR1 was imaged in the contiguous section (section 2), the *indicates CV. **(B)** Representative pseudocolor flow cytometry plots of intrahepatic IBA1^+^ myeloid cells at 0 h vs. 48 h post-CCl_4_. Cells were gated on CD11b^+^ Ly6G^-^ cells. The numbers next to the plots represent frequencies in this gate. **(C)** Representative digital alignment of IF images from serial sections showing on the left GATA6 (yellow), as a marker of peritoneal macrophages, and on the right IBA1 (red). The demarcation (white line) is to facilitate the visual merge. DAPI nuclei are in blue, scale bar =25 μm. The perimeter of the CV is delineated in white. N=4 mice per group.

The kinetics of IBA1^+^CLEC4F^-^ macrophages in the tissue as determined by mIF (**Figure 4G**), mirrored the kinetics of Ly6C^+^ CX3CR1^+^ MoMFs as determined by flow cytometry (**Figure 3F**). In addition, IBA1^+^CLEC4F^-^ macrophages did not express the KC marker CLEC4F and were absent in the non-injured liver (**Figures 4G, H**) (5). Consequently, we hypothesized that the IBA1^+^CLEC4F^-^ macrophages are MoMFs specially recruited to the necrotic tissue in response to injury. Indeed, our additional mIF analysis of liver tissues demonstrated that IBA1^+^CLEC4F^-^ cells recruited to the necrotic/inflammatory area around CV at 48 h post-CCl_4_ expressed CX3CR1 and the monocytic marker CCR2 (**Figure 6A**). Consistent with this, our flow cytometry analysis of intrahepatic myeloid IBA1^+^ cells showed that this population is expanded at 48 h post-CCl_4_ compared to controls and expressed the MoMF-associated markers, Ly6C and CX3CR1, as well as the proliferation marker Ki67 (**Figure 6B**) (5, 6). To rule out the possibility that these IBA1^+^CLEC4F^-^ macrophages are of peritoneal origin, we assessed their GATA6 expression, given that F4/80^hi^ GATA6^+^ peritoneal macrophages could infiltrate the liver and locate to the centrilobular area in the CCl_4_ model (3). Serial labelling and digital tissue alignment of GATA6 and IBA1 staining showed that most CV-associated IBA1^+^ macrophages do not express GATA6 (**Figure 6C**). Collectively, these observations strongly suggest that IBA1^+^CLEC4F^-^ macrophages are MoMFs, which are recruited to the necrotic/inflammatory area around CV in response to injury.

### CLEC4F^+^ KCs of yolk sac origin replenish the hepatic macrophage pool during tissue repair

**Figure 7:**
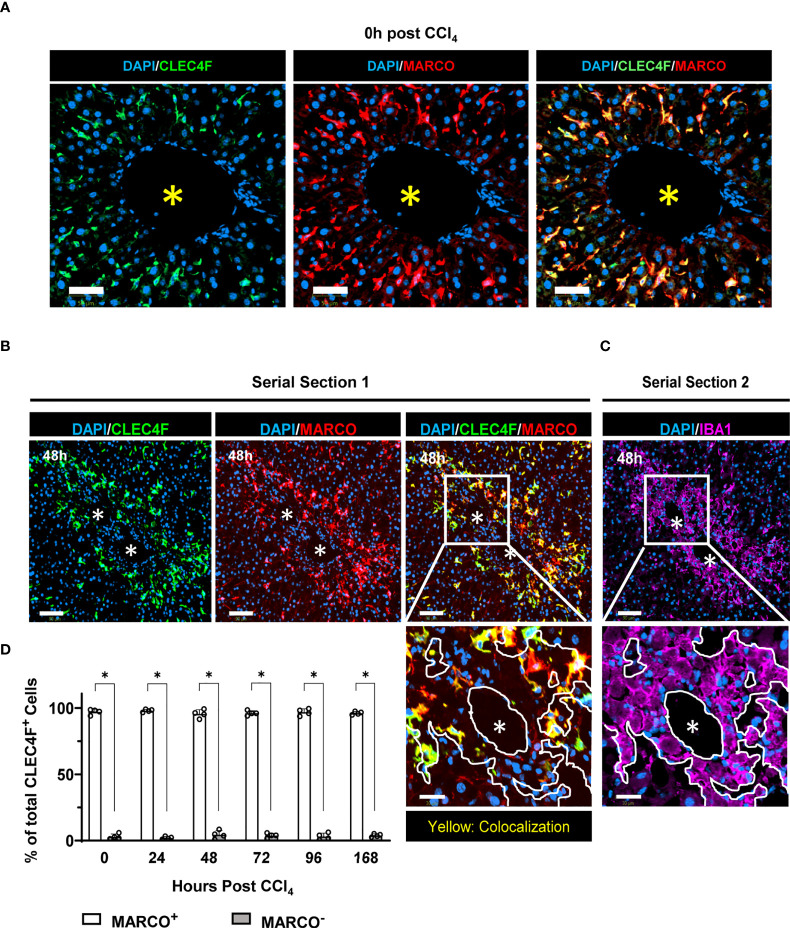
CLEC4F^+^ KCs of yolk sac origin replenish the hepatic macrophage pool during tissue repair. **(A)** Representative mIF images from liver sections at the steady state (0 h), CLEC4F in green (left), MARCO in red (middle) and merge (right). Nuclei counterstained with DAPI (blue), scale bar 50 μm. **(B)** Representative mIF images from liver sections, CLEC4F in green (left), MARCO in red (middle), and merge (right) on serial section 1. **(C)** IBA1 IF (magenta) on serial section 2 (upper panel), scale bar = 90 μm. The inset shows high magnification of the area delineated in the upper panel, scale bar = 30 μm, * indicates CVs. The demarcation (white lines) delineates the area occupied by IBA1^+^ cells around a CV. **(D)** Quantification of MARCO^+^ vs. MARCO^-^ CLEC4F^+^ KCs. N=4 mice per group. Data are shown as mean ± SEM. Statistical analysis was performed using Mann–Whitney U test. *P < 0.05.

Landmark studies have shown that, under physiological conditions, YS-KCs self-maintain independently of circulating progenitors (7, 12). However, in response to injury, opposing results have been reported as to the capacity of monocyte/MoMFs to differentiate into BM-KCs and replace the dead resident YS-KCs (5, 10, 11, 31). Lineage tracing experiments using the APAP-, highly homologous to ours herein, and the radiation-induced hepatic injury models revealed MARCO as a specific marker of YS-KCs and completely absent on infiltrating monocytes or MoMFs (5, 10). Moreover, MARCO was shown to be the marker defining one of the two major subpopulations of resident hepatic macrophages in humans (32). A further support of this notion is the finding identifying bona fide KCs across species and revealing that MARCO is expressed by KCs in uninjured livers of pigs, macaques, hamsters, chicken, and zebrafish (33). Thus, to identify the origin of CLEC4F^+^ KCs replenishing the KC pool during repair, we used MARCO as the marker of YS-KCs (5, 10, 11). YS-KCs are identified as CLEC4F^+^ MARCO^+^ while BM-KCs are CLEC4F^+^ MARCO^-^. Multiplexing CLEC4F with MARCO showed that CLEC4F^+^ KCs are MARCO^+^ at all time points examined, from steady state (**Figure 7A**) until the late repair phase when the KC pool was replenished (**Figures 7B, D**). This finding suggests that virtually all KCs that repopulated the hepatic tissue during repair are of yolk sac origin. Visual inspection showed that the small percentage of CLEC4F^+^ MARCO^-^ cells (~5%) could be attributed to suboptimal MARCO staining since the signal was weak but present (Data not shown). In addition, we performed digital image alignment of consecutive serial sections and demonstrated that there was no spatial overlap between IBA1^+^ macrophages, in direct contact with CVs, and MARCO^+^ cells suggesting that IBA1^+^CLEC4F^-^ macrophages around CVs are MARCO^-^, and thus are not YS-derived (**Figures 7B, C**, insets), but rather monocyte-derived. In summary, these observations suggest that the original YS-KCs that die during necroinflammation are replaced by proliferation of the remaining YS-KCs (CLEC4F^+^ MARCO^+^) around the CVs.

### IBA1^+^CLEC4F^-^ macrophages are in closer contact with activated hepatic stellate cells than CLEC4F^+^ KCs

**Figure 8:**
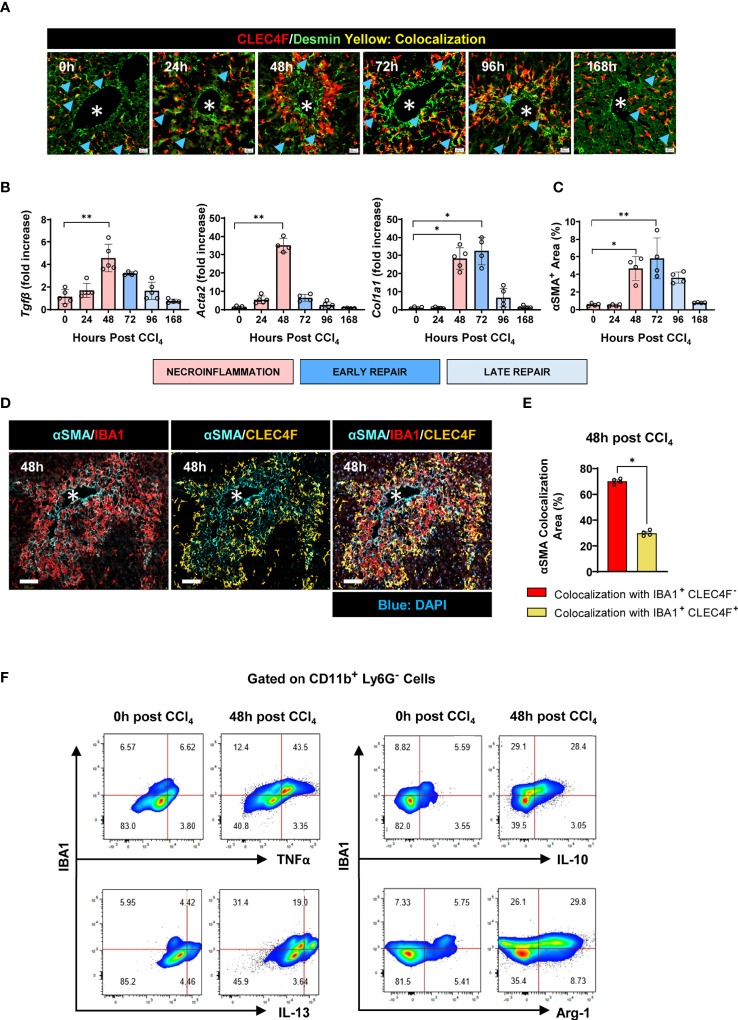
IBA1^+^ CLEC4F^-^ macrophages interact with aHSCs to a greater extent than CLEC4F^+^ KCs. **(A)** Representative mIF images from liver sections showing CLEC4F (red), Desmin (green), and DAPI (blue). Blue arrowheads point at colocalization between CLEC4F and Desmin, scale bar= 20 μm. **(B)** Hepatic mRNA expression of *Tgfb, Acta2*, and *Col1a1*. The mRNA expression data represented fold increase relative to 0 h controls and was normalized to 28s. **(C)** The density of αSMA^+^ HSCs staining in liver sections expressed as % of total tissue area. **(D)** Representative mIF images of IBA1 (red), CLEC4F (yellow), αSMA (cyan) and DAPI (blue), scale bar= 100 μm, * indicates CVs. **(E)** Percentage of colocalization between αSMA^+^ HSCs and either IBA1^+^CLEC4F^-^ cells or CLEC4F^+^ KCs. **(F)** Representative flow cytometry plots of IBA1^+^ intrahepatic myeloid cells isolated from the liver at 0 and 48 h post-CCl_4_ and incubated with BFA and monensin for 6 h prior to staining. The populations shown were pre-gated in CD11b^+^ Ly6G^-^ and the frequencies indicated are relative to this gate. N=4-5 mice per group. Data are shown as mean ± SEM. Statistical analysis was performed using one-way ANOVA followed by Dunn's Multiple Comparison Test **(B, C)** and Mann-Whitney U test **(E)**. *P < 0.05, **P < 0.01.

Activated HSCs (aHSCs) are positive for alpha smooth muscle actin (αSMA^+^) and are the main source of ECM proteins in self-resolving liver inflammation and fibrosis (34, 35). While all HSCs in mice are Desmin^+^, only aHSCs are αSMA^+^ (36). Several studies have supported the notion that both MoMFs and KCs are able to activate HSCs (35). Indeed, 3D reconstruction showed that αSMA^+^ aHSCs wrapped themselves around both CLEC4F^+^ KCs and IBA1^+^CLEC4F^-^ MoMFs suggesting that either macrophage subpopulation could be the source of activating signals (Video 1 in Supplementary information). To further dissect the role of these subsets and evaluate their individual relative contribution to the activation of HSCs and the kinetics involved, we first analyzed the colocalization of CLEC4F^+^ KCs and Desmin^+^ HSCs. We observed that HSCs are in direct contact with resident CLEC4F^+^ KCs in the steady state (**Figure 8A**). Some degree of direct contact was observed at all time points post-CCl_4_ (**Figure 8A**). Since there are no infiltrating IBA1^+^CLEC4F^-^ macrophages in the uninjured liver, we reasoned that the early activating signals of HSCs upon injury are most likely originating from colocalized CLEC4F^+^ KCs.

Next, we established the kinetics of tissue repair as demonstrated by upregulation of the pro-fibrogenic genes *Tgfb*, *Acta2* and *Col1a1* encoding for TGF-β, α smooth muscle actin and collagen, respectively between 48-72 h (**Figure 8B**). This was associated with HSC activation at 48 h post-CCl_4_ as demonstrated by their increased expression of αSMA (**Figure 8C**). At this time point, infiltrating IBA1^+^ CLEC4F^-^ macrophages colocalized with αSMA^+^ aHSCs to a larger extent than resident CLEC4F^+^ KCs, suggesting that this population may be a more significant source of HSC activating signals in the necrotic tissue (**Figures 8D, E**). Furthermore, we characterized the inflammatory and pro-resolving profiles of intrahepatic IBA1^+^ myeloid cells by flow cytometry. Our data show that they exhibit upregulated levels of TNFα, IL-13, IL-10 and Arg-1 at 48 h post-CCl_4_ compared to controls, supporting their potential role in modulating the activation and deactivation states of colocalized HSCs (**Figure 8F**). Collectively, these data suggest that resident CLEC4F^+^ KCs may provide early activating signals to colocalized HSCs, which then become motile and migrate to the CV necrotic area where they interact with IBA1^+^CLEC4F^-^ macrophages to acquire a fully activated phenotype.

## Discussion

While bulk and single cell transcriptomics have provided a wealth of knowledge on the different subpopulations of hepatic macrophages and their phenotypes and roles during homeostasis and disease, their spatial behavior and cell-cell interactions in the tissue remain to be defined. We examined the spatial distribution of subpopulations of liver macrophages and their temporal association with other immune and non-immune hepatic cells in the context of the wound healing response to acute liver injury. Our study is dissecting the kinetics and tissue spatial distribution of hepatic macrophages, namely KCs and MoMFs in response to acute liver injury. Mapping the position of these two subpopulations of macrophages relative to one another was essential for the characterization of their neighboring yet different microanatomical niches, highly suggesting non-overlapping functions for each population.

By examining the immune signatures during the response to an acute liver injury, our flow cytometry analysis showed that liver resident immune populations decreased during necroinflammation but recovered during tissue repair, including KCs, B, T, and NKT cells. Conversely, circulating myeloid cells including neutrophils, monocytes, and MoMFs, massively infiltrated the liver during necroinflammation and progressively declined during repair. We observed two different waves of infiltrating neutrophils during necroinflammation and repair. Since neutrophils do not proliferate in the liver, these two waves are likely the result of two independent recruitment events, probably modulating different effector functions of neutrophils at different phases of the wound healing response to acute liver injury (37). Indeed, while a pro-inflammatory profile has been attributed to neutrophils *via* their production of reactive oxygen species (ROS) and cytokines such IL-1β and TNF-α, a pro-repair function has also been assigned to these cells in their response to liver damage (38–40). The exact role of neutrophils in the different phases of liver wound repair should be defined in future studies.

MoMFs became the predominant phagocyte population during the transition from necroinflammation to early repair, in agreement with the current view that these macrophages are highly plastic cells and experience *in-situ* switching from an inflammatory to a pro-restorative phenotype (6, 10). This observation supports the notion of MoMFs as dual effectors of inflammation and repair and may explain the divergent outcomes of MoMF depletion during necroinflammation versus repair in different injury models (6, 10, 41–43). Using mIF, we identified two subsets of hepatic macrophages: resident CLEC4F^+^ KCs and infiltrating IBA1^+^CLEC4F^-^ macrophages, with multiple evidence indicating that the latter are a subset of MoMFs. Since together CLEC4F^+^ KCs and IBA1^+^CLEC4F^-^ MoMFs occupied most of the necrotic area, they are arguably the predominant macrophage subsets in the response to CCl_4_ toxicity, directly suggesting their functional relevance. By further investigating the kinetics and spatial distribution of these subsets, our data demonstrate that while the KC population was partially depleted during necroinflammation and recovered at repair, MoMFs behaved contrariwise in these disease phases. Previous studies have also described these macrophage populations at the injury site in liver disease (44–46), but at the individual level and without further characterization of their distribution in time and space. Characterization of MoMFs using the acute model of sterile hepatic injury detected the classical CCR2^hi^/CX3CR1^-^ monocytes at the site of injury as early as 8 h post injury. Subsequently, at 48h post injury, these monocytes differentiated into MoMFs in a process that involved upregulation of CX3CR1 and downregulation of CCR2 and LyC. However, this study did not examine the kinetics and distribution of the other major macrophage subpopulation, KCs, and the model used lacked the zonated injury pattern observed in intoxication-induced acute liver injury (6). On the other hand, in the APAP acute injury model, Zigmond et al. used flow cytomtery to show that KCs are depleted during necroinflammation and recover during the resolution/repair phase by self-renewal with no contribution from circulating Ly6C^hi^ inflammatory monocytes (10). Our study expanded these observations by using tissue imaging where we demonstrate the overall spatial behaviour of KCs during the healing response to acute live injury and showed the specific location of KCs around CVs where KCs proliferated during tissue repair. Furthermore, we show that depletion of KCs at the early response phase is followed by the invasion of MoMFs into the site of injury, supporting the notion that signals generated upon activation and depletion of KCs initiate the recruitment of circulatory monocytes and their differentiation into what is known as restorative macrophage population, herein MoMFs (47, 48). Our further spatial evaluation of these subpopulations revealed distinct microanatomical locations, with MoMFs filling the inner necrotic areas and KCs surrounding them. Other papers, rather investigating chronic models of liver injuries, have imaged the spatial behavior of KCs or of MoMFs but at the individual level and with no reference to the disease stage (6, 11, 20, 33, 49). In addition, most of the tissue work done on acute liver injury has relied on markers that do not allow the proper differentiation of the different macrophage subpopulations like MoMFs, peritoneal macrophages, liver capsular macrophages and resident KCs (e.g., F4/80) (10, 20), while we used in our study more specific markers defined using multiomic technologies (e.g., CLEC4F, GATA6, MARCO) (2, 5, 10, 14). Thus, our observations expand knowledge as to the contribution of hepatic macrophages to liver injury by showing that, during repair, CLEC4F^+^ KCs proliferated around healed CVs from where they seemed to have radiated outwards and colonized the partially depleted surrounding areas. Steady state KCs, broadly assumed to be sessile, become thus motile during late repair to move from the CV-associated clusters to the surrounding sinusoidal regions and re-establish the homeostatic density (9, 45).

Fate mapping approaches have revealed that resident YS-KCs persist in the tissue by self-renewal, independently of monocytic hematopoietic progenitors in the steady state (4, 7). However, contradictory reports exist on the capacity of inflammatory monocytes/MoMFs to differentiate into tissue-resident macrophages (BM-KCs) in response to injury and replace the original YS-KCs (5, 10, 11, 14, 31). Here, we demonstrated that CLEC4F^+^ KCs that repopulated the liver during tissue repair were yolk sac derived, while MoMFs were not. This suggests that even in the presence of significant tissue damage and KC death, the remaining original YS-KCs retained an advantage over other macrophage population in terms of replenishing the KC pool. Our results support the emergent view that the generation of BM-KCs depends on niche availability, which is not equal in different injury models, and only models with extensive KC depletion make the niche available for engraftment of infiltrating monocytes/MoMFs (5, 10, 11, 14, 31). Indeed, repopulation of KCs in the KC-Diphteria toxin receptor (DTR) mouse model involved the contribution of circulatory monocytes (49). Diphteria toxin in such model induced 100% depletion of KCs so there are no KCs left to replenish the niche. On the other hand and in accordance with our findings, the APAP liver injury model, where KCs were partially depleted, lineage tracing showed that KCs of yolk sac origin replenish the KC compartment independently of bone marrow-derived cells (10).

Activation of HSCs is a major step in the repair processes during acute liver injury and to fibrosis development under chronic injuries (50, 51). Activated HSCs assume a myofibroblast phenotype, upregulating expression of αSMA and releasing extracellular matrix proteins, including collagen (52). Previous studies have found that both KCs and MoMFs can activate HSCs, but the relative individual contribution and kinetics are still unknown. We took advantage of simultaneous visualization of CLEC4F^+^ KCs and IBA1^+^CLEC4F^-^ MoMFs to gain insight into the individual contribution of these subsets to HSC activation. We demonstrated that quiescent Desmin^+^αSMA^-^ HSCs were in direct contact with KCs in the steady state making the resident macrophages the most likely source of initial activating signals upon damage as previously reported (49). Indeed, KCs are important activators of HSCs *via* the release of pro-inflammatory mediators and growth factors (44, 53). Through their release of reactive oxygen species, and IL-6 induction, KCs were shown to contribute to HSC activation and fibrogenic differentiation (54). KCs are also one of the sources of the major fibrogenic cytokine TGF-β (55, 56). Indeed, our data also demonstrated upregulation of TGF-β concomitantly with an activated state of HSCs. As the response to injury progressed, αSMA^+^ aHSCs further colocalized with MoMFs as compared to KCs, in line with previous work showing that recruited macrophages also contribute to activation of HSCs and, ultimately, fibrosis (57). These results are consistent with a division of labor between these macrophage subsets, where KCs provide early activating signals to colocalized HSCs that may induce their motility and migration into the necrotic zone. Once in this region, monocyte-derived macrophages are highly likely the major source of the remaining signals that induce the fully activated phenotype observed at 48 h post-CCl_4_.

In summary, we used the well established CCl_4_ model of acute liver injury that exhibits important features of the human response leading to inflammation and fibrosis. We show that CLEC4F^+^ KCs and IBA1^+^CLEC4F^-^ MoMFs are the predominant hepatic macrophages during CCl_4_-mediated acute liver injury. These two subpopulations exhibited different origins and morphology, infiltrated the necrotic area at different times, and occupied neighbouring but unique microanatomical locations. KCs proliferated around CVs and colonized the surrounding tissue, whereas infiltrating MoMFs was transient and did not contribute to the replenishment of the KC pool in the liver. Lastly, while KCs colocalized with HSCs in steady state conditions and may be the source of early activating signals, MoMFs were likely responsible for activating HSCs during the transition from necroinflammation to repair, enhancing their pro-repair functions and initiating the healing process. Future studies investigating the underlying mechanism(s) and using a more physiologically relevant model are warranted.

## Data availability statement

The original contributions presented in the study are included in the article/**Supplementary Material**. Further inquiries can be directed to the corresponding author.

## Ethics statement

The animal study was reviewed and approved by Institutional Animal Care and Use Committee-CRCHUM.

## Author contributions

MFM substantially contributed to the conception and design of the study, the undertaking of experiments, and the acquisition, analysis, and interpretation of data. He drafted the article and participated in its critical revision for important intellectual content. MNA, SMa, SMu, and DOL contributed to the undertaking of experiments and the acquisition, analysis, and interpretation of data. DV-B, VT, NB and AD contributed to the acquisition, analysis, and interpretation of data. GH contributed to writing the article and revising it critically for important intellectual content. NHS, the corresponding author, substantially contributed to the conception and design of the study and the critical revision of the article for important intellectual content. She is responsible for the acquisition of funding. All authors approved the final version of the manuscript. All authors contributed to the article and approved the submitted version.

## Funding

This study was funded by research grants from the Canadian Liver Foundation and the Canadian Institutes of Health Research (CIHR) (PJ4-169659 and PJT-175134). MFM received a doctoral fellowship from the Fonds de Recherche du Quebec-Santé (FRQ-S). MNA received the bourse d’exemption des droits de scolarité supplémentaires from the Université de Montréal and a doctoral fellowship from the Canadian Network on Hepatitis C (CanHepC). CanHepC is funded by a joint initiative of the CIHR and the Public Health Agency of Canada (HPC-178912). SMa received a doctoral fellowship from CanHepC.

## Acknowledgments

We acknowledge the following CRCHUM platforms for excellent technical assistance: Animal facility, molecular pathology, microscopy and flow cytometry. We also thank Rainer Gangnus and Regan Baird from Visiopharm for their technical insights and assistance.

## Conflict of interest

The authors declare that the research was conducted in the absence of any commercial or financial relationships that could be construed as a potential conflict of interest.

## Publisher’s note

All claims expressed in this article are solely those of the authors and do not necessarily represent those of their affiliated organizations, or those of the publisher, the editors and the reviewers. Any product that may be evaluated in this article, or claim that may be made by its manufacturer, is not guaranteed or endorsed by the publisher.

## Supplementary material

SUPPLEMENTARY TABLE S1 Immunofluorescence antibodies and reagents.

SUPPLEMENTARY TABLE S2 Flowcytometry antibodies and reagents for all other techniques.

SUPPLEMENTARY TABLE S3 Sequence of Primers.

SUPPLEMENTARY FIGURE S1 Ki67 levels are highest at 48 h post-CCl_4_. Representative Ki67 IHC images at 0, 24, 48, 72, 96 and 168 hours post -CCl_4_, scale bar = 200 μm.

SUPPLEMENTARY FIGURE S2 Circulating myeloid cells are recruited to the liver in response to CCl_4_ acute injury during the necroinflammatory and tissue repair phases. **(A)** Gating strategy for intrahepatic phagocyte. **(B)** Representative pseudocolor plots of the kinetics of major phagocyte populations induced in response to CCl_4_ injury. N=4-5 mice per group.

SUPPLEMENTARY FIGURE S3 Recovered CD11b^+^ MHCII^+^ CD64^+^ F4/80^+^ cells express CX3CR1, and therefore are not KCs. **(A)** Gating strategy to define KCs from total intrahepatic leukocytes. **(B)** Contour plots showing CX3CR1 expression by total myeloid cells (left column), by CD11b^+^ MHCII^+^ CD64^+^ F4/80^+^ cells (center column), and the merged overlay (right column).

SUPPLEMENTARY FIGURE S4 IBA1^+^CLEC4F^-^ macrophages and CLEC4F^+^ KCs exhibit different spatial profiles in response to CCl_4_-induced acute liver injury. **(A)** Representative IBA1 (red) and CLEC4F (yellow) IF images of whole tissue at 48 hours post CCl_4_ at low magnification, scale bar = 1 mm. **(B)** High magnification of inset from A) showing DAPI/IBA1 (left), DAPI/CLEC4F (center), and merge (right), scale bar =100 μm.

SUPPLEMENTARY FIGURE S5 IBA1^+^ CLEC4F^-^ macrophages and IBA1^+^ CLEC4F^+^ KCs are present in murine fibrotic liver. **(A)** Multiplex IF showing IBA1 (red) and CLEC4F (green) in liver sections from 12 weeks CCl_4_- treated mouse. Colocalization of red and green shows as yellow, scale bar = 200 μm, * designates CVs. **(B)** Higher magnifications of insets I, II, and III from **(A)**. IBA1/DAPI (left column), CLEC4F/DAPI (center column), and merge (right column). White arrowheads pointing to IBA1^+^ CLEC4F^-^ macrophages (red) and cyan arrowheads pointing at IBA1^+^ CLEC4F^+^ KCs (green-yellow), scale bar = 100 μm.

SUPPLEMENTARY FIGURE S6 Globular shaped IBA1^+^ cells infiltrate the necrotic tissue at 24 h and replace hepatocytes in the area surrounding CVs by 48 h post-CCl_4_. Aligned serial sections of hepatic tissue: section 1 (H&E) and section 2 (IBA1 IHC). Red arrowheads pointing at infiltrating amoeboid IBA1^+^ macrophages at 24 h post-CCl_4_, scale bar= 50 μm. Yellow arrowheads pointing at IBA1^+^ macrophages at 48 post-CCl_4_ with particulate material inside and occupying the previous location of hepatocytes around CVs, scale bar= 20 μm (insets). N=4 mice per group.

SUPPLEMENTARY VIDEO 1 Activated hepatic stellate cells, IBA1^+^CLEC4F^-^ macrophages and CLEC4F^+^ KCs establish direct contact around injured central veins at 48 h post-CCl_4_. Three-dimensional (3D) reconstruction of the interaction between an activated HSC in red (based on αSMA signal), an IBA1^+^CLEC4F^-^ macrophage in yellow (based on IBA1 signal), and a CLEC4F^+^ KC in white and green (based on CLEC4F signal in white). Images or z-stacks were acquired using ×40 objective, ApoTome 2, Zeiss. Three-dimensional reconstruction was done using Imaris 8.1.2 software (Bitplane, Zurich, Switzerland).

## Abbreviations

ALT, alanine aminotransferase; αSMA, alpha smooth muscle actin; BFA, brefeldin A; CCl_4_ carbon tetrachloride; CCL2, C-C motif chemokine ligand 2; CCL7, C-C motif chemokine ligand 7; CCR2, C-C motif chemokine receptor 2; CV, central vein; CX3CR1, CX3C chemokine receptor 1; ECM, extracellular matrix; FlowSOM, Flow with Self Organizing Maps; HSC, hepatic stellate cell; IHL, intrahepatic leukocyte; IL-, interleukin-; KC, Kupffer cells; Ly6C, lymphocyte antigen 6 complex, locus C; Ly6G, lymphocyte antigen 6 complex, locus G6D; M-CSF, macrophage colony stimulating factor; mIF, Multiplex Immunofluorescence; MoMFs, monocyte-derived macrophages; MARCO, macrophage receptor with collagenous domain; NCMs, non-classical monocytes; NKT, natural killer T cell; TGFβ, transforming growth factor-β; TNFα, tumor necrosis factor-α; t-SNE, t-distributed stochastic neighbor embedding analysis; YS, yolk sac.
