# Supplementary material for: Distinct spatial distribution and roles of Kupffer cells and monocyte-derived macrophages in mouse acute liver injury
Source: Front Immunol. 2022 Sep 30;13:994480. doi: 10.3389/fimmu.2022.994480 (PMC9562324; doi:10.3389/fimmu.2022.994480)
Supplement: SUPPLEMENTARY TABLE S1 — Immunofluorescence antibodies and reagents. [file Table_1.pdf]

Table S1. Immunofluorescence antibodies and reagents

Immunohistochemistry Abs	Company	Clone	Catalog #	State,Country
Ki67	Invitrogen	SP6	MA5-14520	Canada
IBA1	Fujifilm WAKO Chemicals	Rabbit Polyclonal	019-19741	USA
**Immunofluorescence Primary Abs**				
CLEC4F	R&D Systems	Goat Polyclonal	AF2784	Canada
IBA1	Fujifilm WAKO Chemicals	Rabbit Polyclonal	019-19741	USA
MARCO	Abcam	EPR22944-64	ab239369	Canada
αSMA	Sigma Aldrich	1A4	A2547-100UL	Canada
CCR2	R&D Systems	475303	MAB55382-SP	Canada
CX3CR1	Thermofisher Scientific	1H14L7	702321	Canada
Desmin	Invitrogen	Rabbit Polyclonal	PA5-16705	Canada
GATA-6	Cell Signaling	D61E4	5851T	Ontario, Canada
**Immunofluorescence Secondary Abs**				
Donkey anti-goat A568	Invitrogen	Donkey Polyclonal	A-11057	Canada
Donkey anti-rabbit A647	Invitrogen	Donkey Polyclonal	A-31573	Canada
Donkey anti-rabbit A488	Invitrogen	Donkey Polyclonal	A-21206	Canada
Donkey anti-mouse A488	Invitrogen	Donkey Polyclonal	A-21202	Canada
Chicken anti-rat A647	Invitrogen	Chicken Polyclonal	A-21472	Canada
**Other Reagents for Immunofluorescence**				
SlowFade Gold antifade with DAPI	Invitrogen		S36938	Canada
Bovine serum albumin (BSA)	Multicell		800-095-EG	Qc, Canada
Human Serum	Gemini		22210	USA
Sodium Citrate Dihydrate	Millipore Sigma		1545801	Canada
Triton X-100	Sigma Aldrich		T8787-50ML	Canada

Tris-HCl	BioShop		77-86-1	Canada
Tween 20	Fisher Scientific		BP337-500	Canada
Tissufix	Chaptec		T-50	Montreal, Canada
**Solutions for Immunofluorescence**	**Composition**		S36938	
Blocking solution for immunofluorescence	1 % BSA, 10 % human serum, 10 % donkey serum, 0.1 % Tween 20 and 0.3% Triton in PBS			
Glycine solution for saturation of aldehyde groups	Glycine 0,1 M in PBS			
Antigen retrieval solution	Sodium Citrate Buffer (10 mM Sodium Citrate, 0.05% v/v Tween 20, pH 6.0)			
Washing Solution	PBS 0.1% Tween			
