# Supplementary material for: Distinct spatial distribution and roles of Kupffer cells and monocyte-derived macrophages in mouse acute liver injury
Source: Front Immunol. 2022 Sep 30;13:994480. doi: 10.3389/fimmu.2022.994480 (PMC9562324; doi:10.3389/fimmu.2022.994480)
Supplement: SUPPLEMENTARY TABLE S2 — Flowcytometry antibodies and reagents for all other techniques. [file Table_2.pdf]

Table S2. Flowcytometry Antibodies and reagents for all other techniques

Flow Cytometry Abs	Company	Clone	Catalog #	State, Country
CD45 SB645	eBioscience	30-F11	64-0451-82	Canada
CD45 BV650	Biolegend	30-F11	103151	USA
CD11b BV 421	BD Biosciences	M1/70	562605	USA
Ly6C PE-CF594	BD Biosciences	AL-21	562728	USA
Ly6G A488	Biolegend	1A8	127626	USA
Gr-1 A488 (Ly6G/Ly6C)	eBioscience	RB6-8C5	53-5931-82	Canada
IBA1/A1F1 A488	Millipore	20A12.1	MABN92-AF488	Canada
CCR2 PE	R&D Systems	475301	FAB5538P	Canada
CD64 APCe780	eBioscience	X54-5/7.1	212838	Canada
F4/80 BUV395	BD Biosciences	T45-2342	565614	USA
CX3CR1 BV785	Biolegend	SA011F11	149029	USA
MHC II (I-A/I-E) BUV 737	BD Biosciences	M5/114,15,2	748845	USA
Arg-1 PE	eBioscience	A1exF5	12-3697-82	Canada
TNFα PE Cy7	BD Biosciences	MP6-XT22	557644	USA
IL-10 BUV 421	Biolegend	JES5-16E3	505022	USA
IL-13 PE Cy5.5	Novus Biologicals	13A	NBP1-43239PECY55	Canada
CD3 BUV 395	BD Biosciences	145-2C11	563565	USA
CD4 BUV 496	BD Biosciences	GK1.5	564667	USA
CD8 BUV 737	BD Biosciences	53-6.7	564297	USA
CD19 APC H7	BD Biosciences	1D3	560245	USA
NK1.1	Biolegend	PK136	108718	USA
TCRγδ	BD Biosciences	GL3	562892	USA
**Other Flow Cytometry Reagents**				
LIVE/DEAD™ Fixable Aqua Dead Cell Stain Kit	Thermofisher Scientific		L34957	Canada
Brilliant Stain Buffer	BD Horizon		566349	USA
BFA	Sigma Aldrich		B6542	Canada
Monensin Sodium Salt	Sigma Aldrich		M5273	Canada
Foxp3 / Transcription Factor Staining Buffer Set	Thermofisher Scientific		00-5523-00	Canada
**Reagents for RNA Isolation and RT-PCR**				
RNeasy Plus Micro Kit (50)	Qiagen		74034	USA
Transcriptor Universal cDNA Master	Roche		05893151001	Canada
LightCycler® 480 SYBR Green I Master	Roche		4707516001	Canada
**H&E Reagents**				
Eosin	Leica Biosystems		3801600	USA

Hematoxylin Stain Sol., Gil 1. Formulation, Reg.	Ricca Chemical Comp.		3535-32	USA
**Reagents for Isolation of IHLs**				
Benzonase	Millipore Sigma		70664	Canada
Collagenase D	Roche		11088866001	Canada
RPMI 1640	Thermofisher Scientific		11875093	Canada
FBS	Thermofisher Scientific		A31607	Canada
Percoll	Sigma Aldrich		P1644	Canada
Sodium Azide	Fisher Scientific		S-227	Canada
Normal Mouse Serum	Thermofisher Scientific		10410	Canada
Formaldehyde	Sigma Aldrich		F8775	Canada
