# Supplementary material for: Distinct spatial distribution and roles of Kupffer cells and monocyte-derived macrophages in mouse acute liver injury
Source: Front Immunol. 2022 Sep 30;13:994480. doi: 10.3389/fimmu.2022.994480 (PMC9562324; doi:10.3389/fimmu.2022.994480)
Supplement: SUPPLEMENTARY TABLE S3 — Sequence of Primers. [file Table_3.pdf]

Table S3. Primers

**Primers**	**Forward**	**Reverse**
TNF-a	5′-ACTCCAGGCGGTGCCTATGT-3′	5′-GTGAGGGTCTGGGCCATAGAA-3′
IL-1B	5′-GGCAGGCAGTATCACTCATT-3′	5′-GAGGATGGGCTCTTCTTCAAA-3′
CX3CL-1	5′-CGCGTTCTTCCAATTTGTGTA-3′	5′-CTGTGTCGTCTCCAGGACAA-3′
CX3CR1	5′-ATTCTTCATCACCGTCATCAG-3′	5′-ACTAATGGTGACACCGTGCT-3′
CCL2	5′-TCTGGACCCATTCCTTCTTGG-3′	5′-TCAGCCAGATGCAGTTAACGC-3′
CCL7	5′-GGCCTCCTCAACCCACTTCT-3′	5′-CCCTGGGAAGCTGTTATCTTCA-3′
M-CSF	5′-CAGCTGCTTCACCAAGGACT-3′	5′-TCATGGAAAGTTCGGACACA-3′
mCLEC4F	5′-CTTCGGGGAAGCAACAACTC-3′	5′-CAAGCAACTGCACCAGAGAAC-3′
28s	5′-CGAGATTCCCACTGTCCCTA-3′	5′-GGGGCCTCCCACTTATTCTA-3′
**Primers**	**Company**	**NCBI reference**
Col1a1	Qiagen	(NM_007742)
Acta2	Qiagen	(NM_007392)
TGF-β1	Qiagen	(NM_011577)
