# Supplementary figures and images for: Distinct spatial distribution and roles of Kupffer cells and monocyte-derived macrophages in mouse acute liver injury
Source: Front Immunol. 2022 Sep 30;13:994480. doi: 10.3389/fimmu.2022.994480 (PMC9562324; doi:10.3389/fimmu.2022.994480)

**A****Ki67 IHC**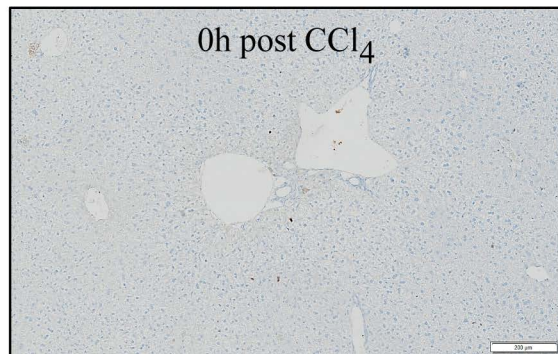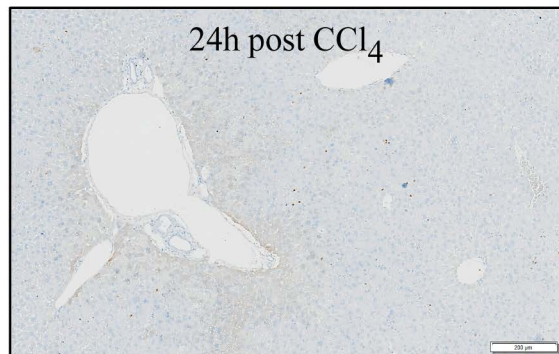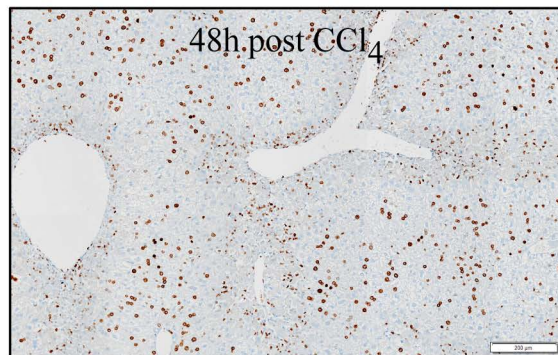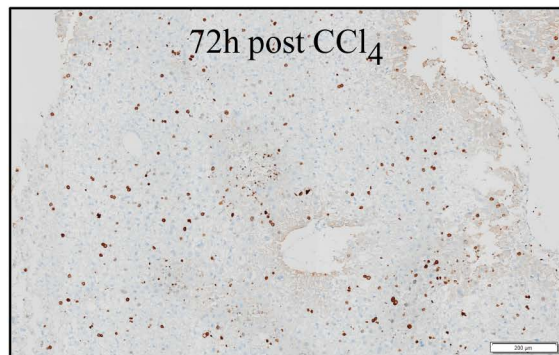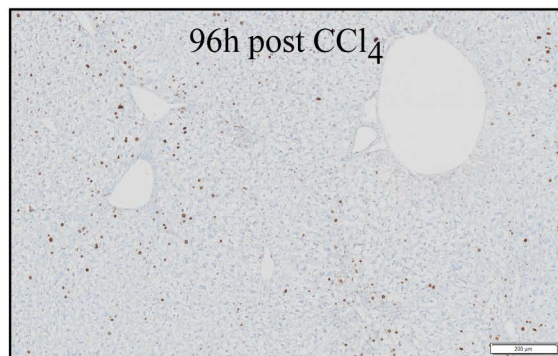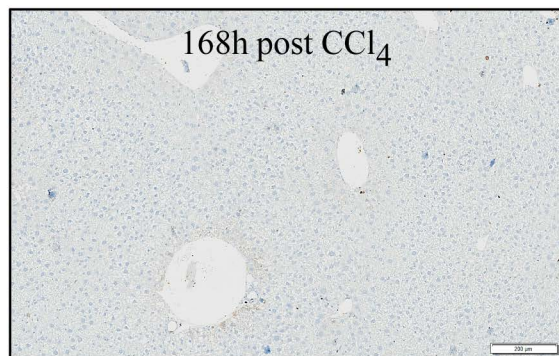

Supplement: SUPPLEMENTARY FIGURE S1 — Ki67 levels are highest at 48 h post-CCl4. Representative Ki67 IHC images at 0, 24, 48, 72, 96 and 168 hours post -CCl4, scale bar = 200 μm. [file Image_1.pdf]

**A**

48h post CCl<sub>4</sub>  
DAPI IBA1 CLEC4F

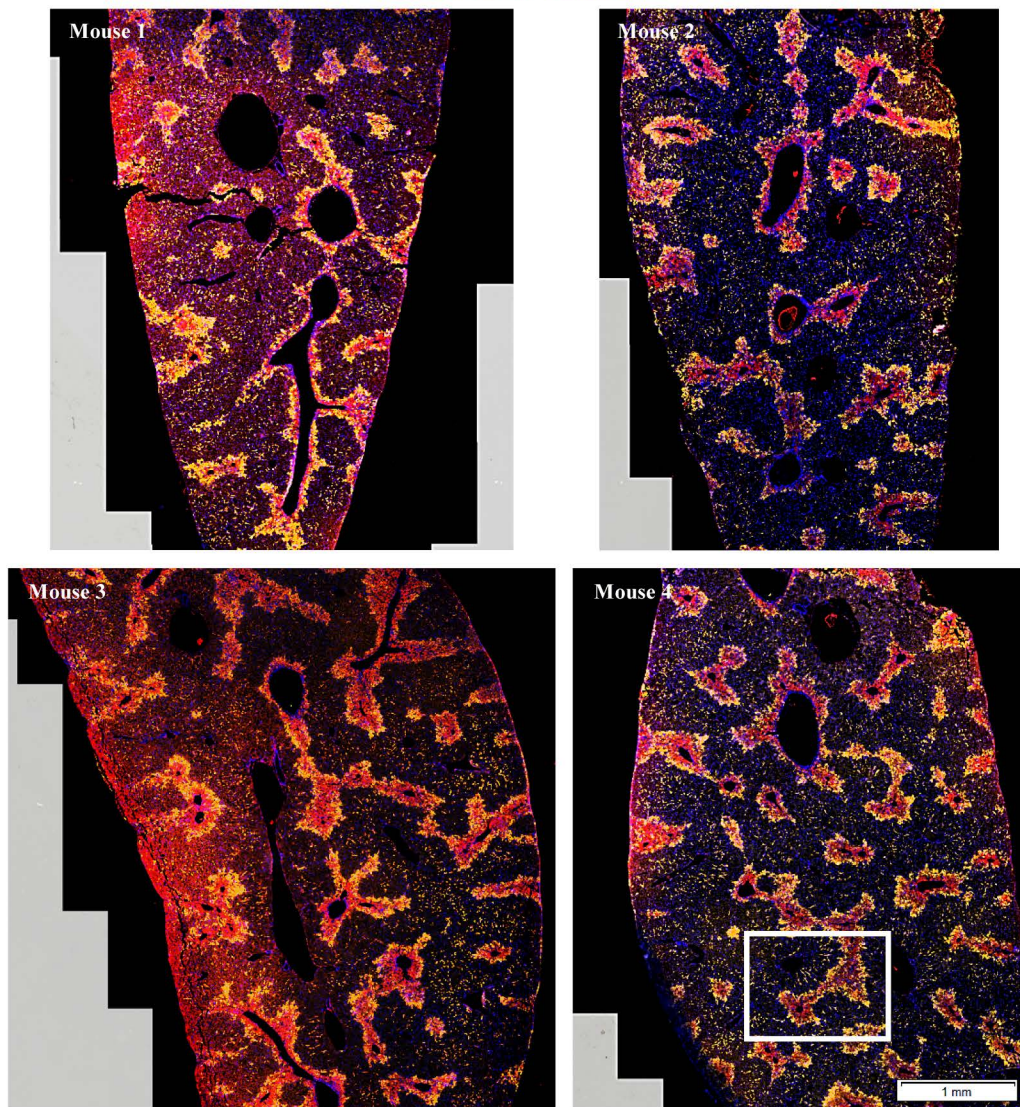**B**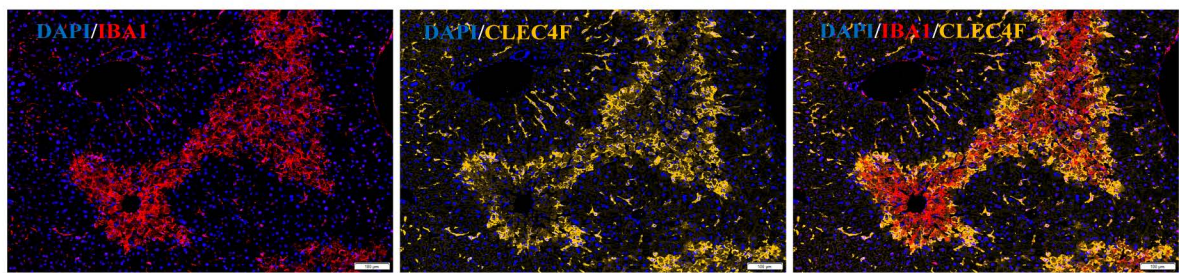

Supplement: SUPPLEMENTARY FIGURE S4 — IBA1+CLEC4F- macrophages and CLEC4F+ KCs exhibit different spatial profiles in response to CCl4-induced acute liver injury. (A) Representative IBA1 (red) and CLEC4F (yellow) IF images of whole tissue at 48 hours post CCl4 at low magnification, scale bar = 1 mm. (B) High magnification of inset from A) showing DAPI/IBA1 (left), DAPI/CLEC4F (center), and merge (right), scale bar =100 μm. [file Image_4.pdf]

A

12 weeks CCl<sub>4</sub>  
DAPI IBA1 CLEC4F

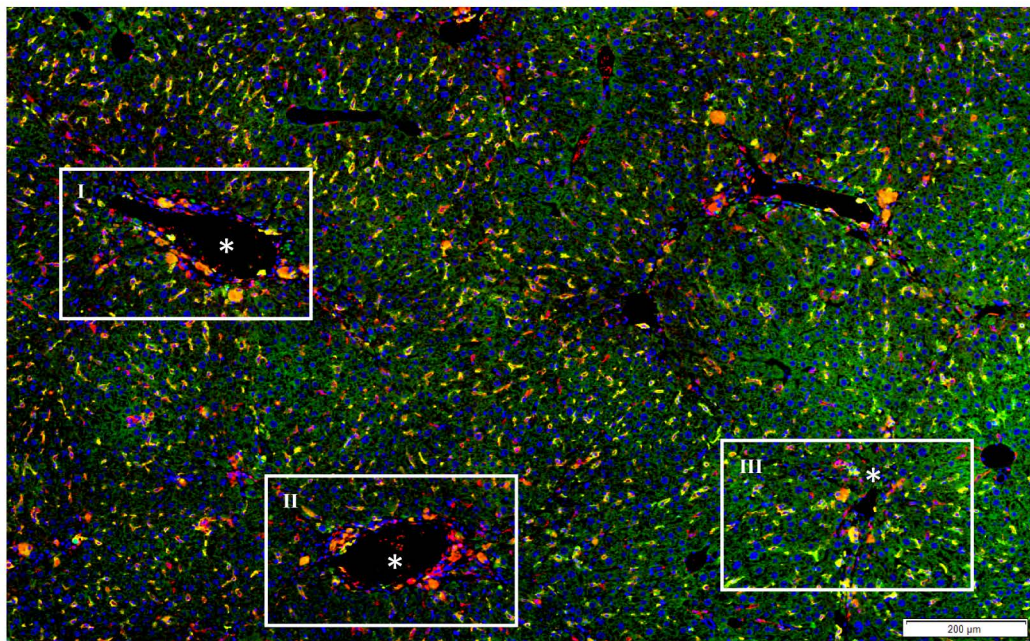

B

DAPI/IBA1

DAPI/CLEC4F

DAPI/IBA1/CLEC4F

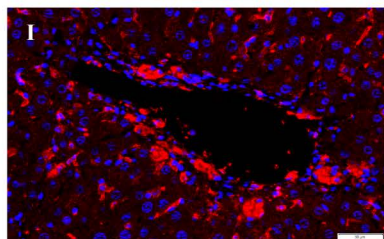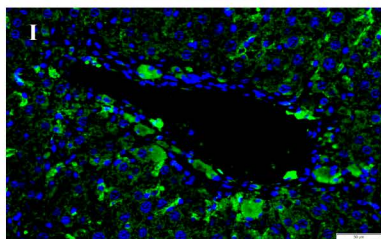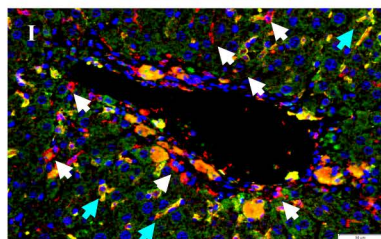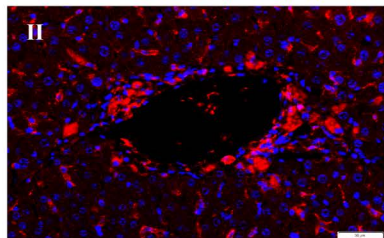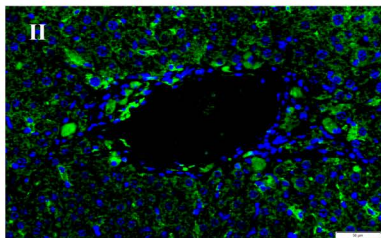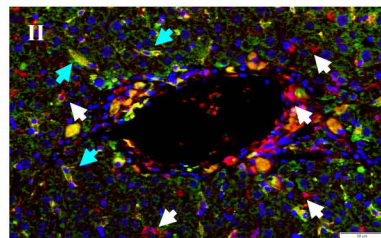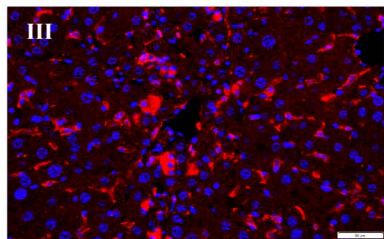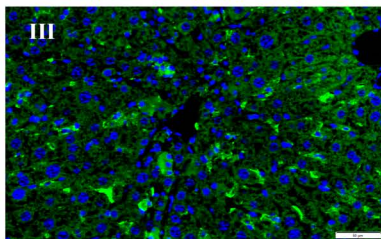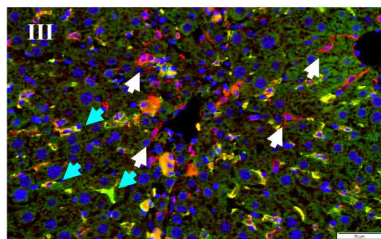

Supplement: SUPPLEMENTARY FIGURE S5 — IBA1+ CLEC4F- macrophages and IBA1+ CLEC4F+ KCs are present in murine fibrotic liver. (A) Multiplex IF showing IBA1 (red) and CLEC4F (green) in liver sections from 12 weeks CCl4- treated mouse. Colocalization of red and green shows as yellow, scale bar = 200 μm, * designates CVs. (B) Higher magnifications of insets I, II, and III from (A). IBA1/DAPI (left column), CLEC4F/DAPI (center column), and merge (right column). White arrowheads pointing to IBA1+ CLEC4F- macrophages (red) and cyan arrowheads pointing at IBA1+ CLEC4F+ KCs (green-yellow), scale bar = 100 μm. [file Image_5.pdf]

H&amp;E Section 1

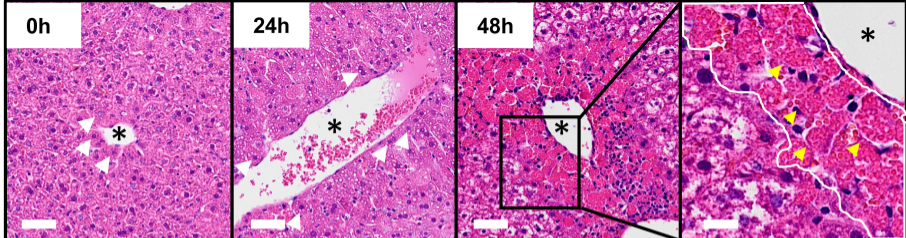

IBA1 Section 2

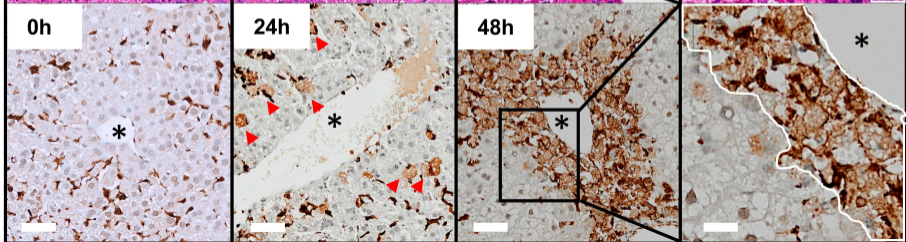

Supplement: SUPPLEMENTARY FIGURE S6 — Globular shaped IBA1+ cells infiltrate the necrotic tissue at 24 h and replace hepatocytes in the area surrounding CVs by 48 h post-CCl4. Aligned serial sections of hepatic tissue: section 1 (H&E) and section 2 (IBA1 IHC). Red arrowheads pointing at infiltrating amoeboid IBA1+ macrophages at 24 h post-CCl4, scale bar= 50 μm. Yellow arrowheads pointing at IBA1+ macrophages at 48 post-CCl4 with particulate material inside and occupying the previous location of hepatocytes around CVs, scale bar= 20 μm (insets). N=4 mice per group. [file Image_6.pdf]
