# Supplementary material for: Distinct spatial distribution and roles of Kupffer cells and monocyte-derived macrophages in mouse acute liver injury
Source: Front Immunol. 2022 Sep 30;13:994480. doi: 10.3389/fimmu.2022.994480 (PMC9562324; doi:10.3389/fimmu.2022.994480)
Supplement: SUPPLEMENTARY FIGURE S2 — Circulating myeloid cells are recruited to the liver in response to CCl4 acute injury during the necroinflammatory and tissue repair phases. (A) Gating strategy for intrahepatic phagocyte. (B) Representative pseudocolor plots of the kinetics of major phagocyte populations induced in response to CCl4 injury. N=4-5 mice per group. [file Image_2.pdf]

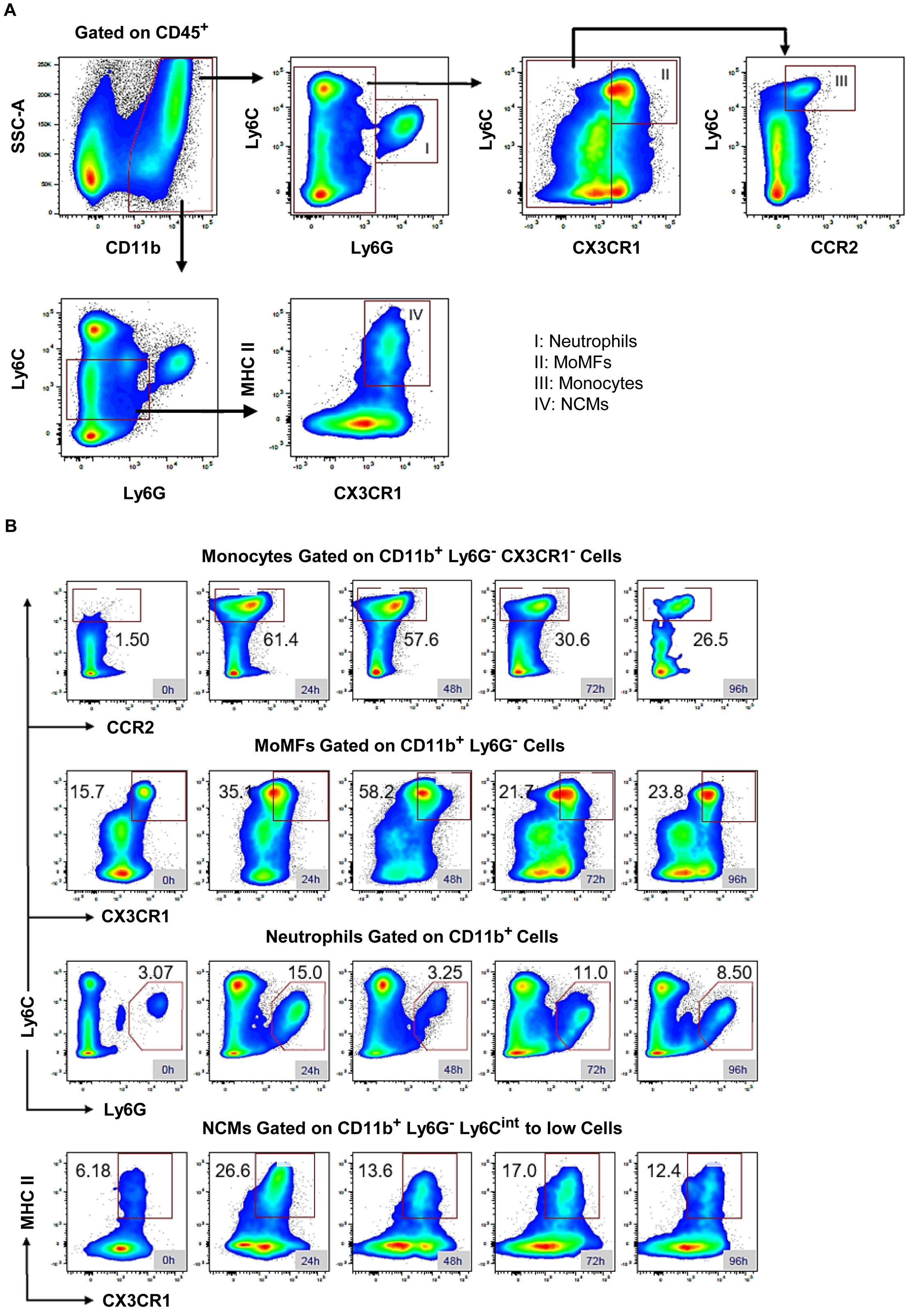

A
Gated on CD45+
SSC-A
CD11b
Ly6C
Ly6G
I
Ly6C
CX3CR1
II
Ly6C
CCR2
III
Ly6C
Ly6G
MHC II
CX3CR1
IV
I: Neutrophils
II: MoMFs
III: Monocytes
IV: NCMs
B
Monocytes Gated on CD11b+ Ly6G- CX3CR1- Cells
1.50
61.4
57.6
30.6
26.5
0h
24h
48h
72h
96h
CCR2
MoMFs Gated on CD11b+ Ly6G- Cells
15.7
35.1
58.2
21.7
23.8
0h
24h
48h
72h
96h
CX3CR1
Neutrophils Gated on CD11b+ Cells
3.07
15.0
3.25
11.0
8.50
0h
24h
48h
72h
96h
Ly6C
Ly6G
NCMs Gated on CD11b+ Ly6G- Ly6Cint to low Cells
6.18
26.6
13.6
17.0
12.4
0h
24h
48h
72h
96h
MHC II
CX3CR1
