# Supplementary material for: Distinct spatial distribution and roles of Kupffer cells and monocyte-derived macrophages in mouse acute liver injury
Source: Front Immunol. 2022 Sep 30;13:994480. doi: 10.3389/fimmu.2022.994480 (PMC9562324; doi:10.3389/fimmu.2022.994480)

A

Gating strategy for exclusion of Ly6Chi monocytes and Ly6G+ neutrophils and gating on the KCs
KCs are CD45+ CD11b+ MHCII+ F4/80+ CD64+ CX3CR1- Ly6C- Ly6G-

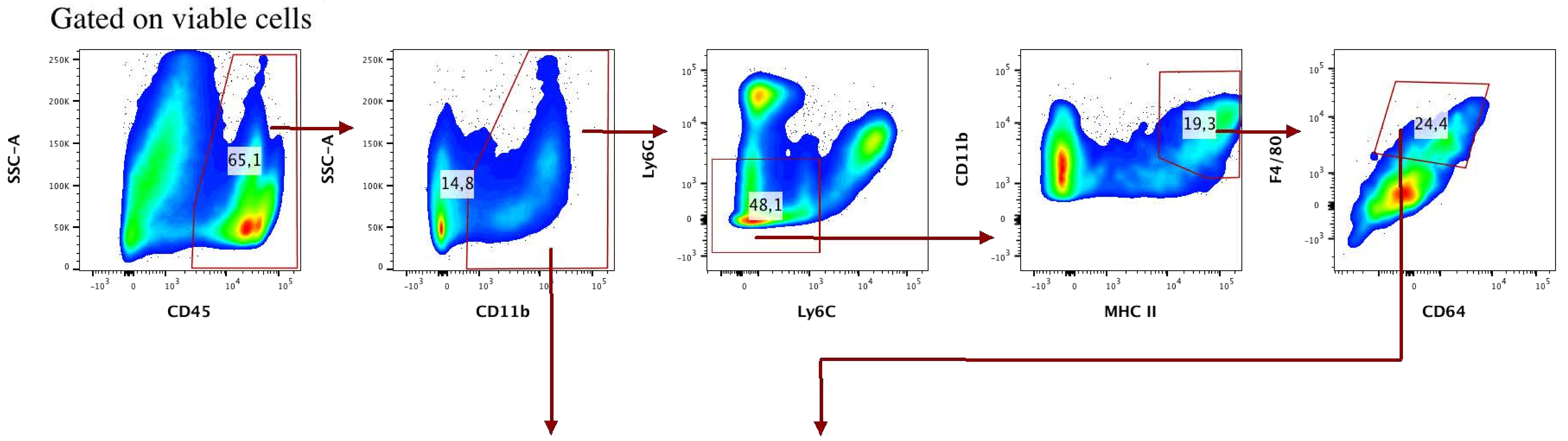

B

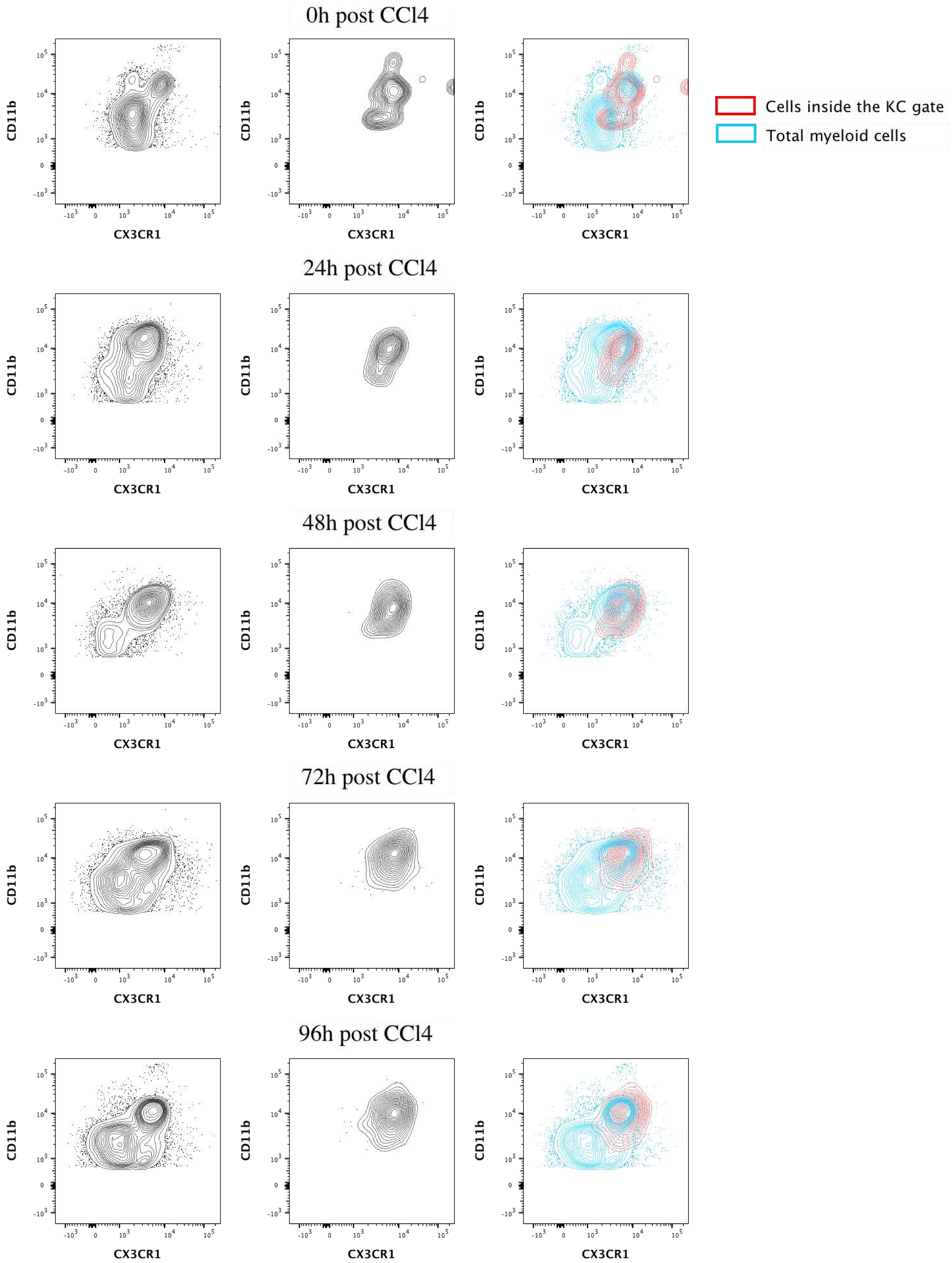

Supplement: SUPPLEMENTARY FIGURE S3 — Recovered CD11b+ MHCII+ CD64+ F4/80+ cells express CX3CR1, and therefore are not KCs. (A) Gating strategy to define KCs from total intrahepatic leukocytes. (B) Contour plots showing CX3CR1 expression by total myeloid cells (left column), by CD11b+ MHCII+ CD64+ F4/80+ cells (center column), and the merged overlay (right column). [file Image_3.pdf]
